# Recent advances and future prospects of the potential-resolved strategy in ratiometric, multiplex, and multicolor electrochemiluminescence analysis

**DOI:** 10.7150/thno.74308

**Published:** 2022-09-21

**Authors:** Shijun Wang, Shu Zhu, Ziqi Kang, Yidan Chen, Xiancheng Liu, Zixin Deng, Kun Hu, Guixue Wang, Yuchan Zhang, Guangchao Zang

**Affiliations:** 1 Institute of Life Science, and Laboratory of Tissue and Cell Biology, Lab Teaching & Management Center, Chongqing Medical University, Chongqing, 400016, China.; 2 Key Laboratory for Biorheological Science and Technology of Ministry of Education, State and Local Joint Engineering Laboratory for Vascular Implants, Bioengineering College of Chongqing University, Chongqing, 400030, China.; 3 Jinfeng Laboratory, Chongqing, 401329, China.

**Keywords:** electrochemiluminescence, potential-resolved strategy, ratiometric electrochemiluminescence sensors, multiplex electrochemiluminescence sensors, multicolor electrochemiluminescence sensors

## Abstract

The potential-resolved strategy has gradually demonstrated its distinct values in electrochemiluminescence (ECL) bio-sensing due to its superior characteristics, such as low instrument requirement, short assay time, and improved sample throughput, in conjunction with spatial- and spectrum-resolved techniques. It has recently been widely generalized into versatile multiple-signal ECL analytic platforms, especially in ratiometric and multiplex ECL sensors, in accordance with some specific principles. Furthermore, luminophore pairs with potential- and wavelength-resolved properties have been utilized to visualize biosensors that display multiple colors depending on analyte concentration. However, only a few comprehensive reports on the principles, construction, and application of various ECL sensors in potential-resolved schemes have been published. This review aims to recount the potential-resolved strategy applying to (a) ratiometric ECL sensors, (b) multiplex ECL sensors, and (c) multicolor ECL sensors and to discuss the distinctions and connections among the application principles of these strategies. Finally, the future prospects of ECL-based potential-resolved analysis are explored.

## Introduction

Electrochemiluminescence (ECL), which combines chemiluminescence and electrochemistry, is the optical emission from the excited luminophores that are produced at the electrode surface via electrochemical high-energy electron transfer reactions 1,2. ECL sensors have recently been widely used in biomedical tests, including tests for drug residues, toxic metal ions, tumor biomarkers, DNA, and even circulating tumor cells and exosomes 2-6 because of their high sensitivity, facile controllability, and simple optical equipment requirements 5,7. However, the majority of the previously reported ECL analysis systems are built on the basis of a single signal output strategy (“signal-on” or “signal-off” mode). This situation restricts the simultaneous detection of multiple biomarkers or the self-calibration of output signals, thus resulting in decreased efficiency, stability, and accuracy 8. Therefore, multisignal output strategies, which exploit multidimensional resolvable signal types and multipass signal accesses 9 and therefore allow for the integration and miniaturization of biosensors that detect multiple targets 10,11, will be a future trend in the research on ECL sensors.

The multiple-signal ECL assay accomplishes its principle by using resolvable signal output probes or by using multichannel detection. Signal-resolving schemes are widely used and can be classified into three categories: spatial-resolved 12,13, spectrum-resolved 14,15, and potential-resolved 16,17. As part of the spatial-resolved technique, segregated sensing arrays on electrode plates are highly sophisticated and exhibit low cross-talk from adjoining sensing zones 18,19. However, the lack of luminous probe pairs with various emission spectra restricts the deployment of the spectrum-resolved ECL method 20. Furthermore, the uneven photon cross-efficiency caused by combining two or more filters to differentiate photons of different wavelengths could reduce the detection sensitivity and accuracy 21,118. In contrast to the latter two modes, the potential-resolved strategy could achieve multisignal outputs in the same working zone by using only a simple potential scan, thus obviating the need for optical instruments and complex electrode construction, greatly simplifying the operation procedure, and reducing analytical time 22. Therefore, compared with the other two strategies, the potential-resolved technique appears to be a more viable and attractive strategy for ECL sensing schemes with multisignal output.

The potential-resolved ECL multisignal strategy is expected to be generalized to versatile sensing platforms, particularly ratiometric ECL sensors and ECL sensors that simultaneously detect multiple markers, for practical biomedical applications. In addition, its use in potential-resolved multicolor ECL (PRMCECL) has grown in popularity in recent years. For this reason, this review provides a comprehensive summary of how the potential-resolved technique is designed and executes its distinctive function with (a) ratiometric ECL sensors, (b) ECL sensors that simultaneously detect multiple markers, and (c) multicolor ECL sensors. Achievements in this area are also discussed. This review also clarifies the distinction and connection among the participation of the three types of ECL sensors in the potential-resolved strategy. Finally, future perspectives for ECL-based potential-resolved analysis are discussed.

## Ratiometric ECL Sensors in the Potential-resolved Strategy

The ECL ratiometric approach uses two distinct signal probes to functionalize the electrode substrate and label material and applies the output signal ratio as the concentration indicator. By self-calibrating two luminescent signals, this dual-response detection strategy can suppress the influence of signal interference, such as microenvironmental pollution 23, from the substrate and the detection system, as well as simplify operation steps 24 and accelerate electron transfer kinetics 25 to improve detection accuracy.

Given the importance of the two ECL output signals with potential-resolvable properties in implementing the ratiometric approach, two rules should be adopted to facilitate ratiometric detection. First, the dual-potential emissions should be composed of two signals that can be distinguished at different excitation potentials 26. In addition, the same co-reactant of luminophores should be used to minimize mutual electrochemical interference 27.

The sensitivity and repeatability of the sensor are highly influenced by the sensor's construction principles 28. Many approaches have been used to achieve dual signal output in practice. Among these approaches, the resonance energy transfer (RET) strategy, co-reactant competition strategy, and internal standard strategy are the most prevalent. New ratiometric designs arose as a result of the complexity of chemical compounds and the limited number of accessible luminescent systems. These designs may be characterized as immunological competition, spatial competition, and enzyme-based ratiometric methods. These new methodologies broaden the scope of feasible resolution solutions and provide novel concepts for multisignal output systems.

### ECL-RET Strategy

The ECL-RET approach involves the energy transfer between a pair of ECL luminophores or between luminophores and intermediate reagents. It generally results in ECL signal conversion from “off-on” to “on-off”. The emergence of RET requires spectrum overlap between the ECL energy donor and the energy acceptor at an appropriate distance 19. ECL-RET has attracted considerable attention as a sensitive and controllable ECL signal regulation strategy for the detection of metals 29-31, proteins 32-34, nucleic acids 35-37, small molecules 38,39, and even cells 40,41. Ideal RET pairs for ratiometric ECL fabrication should present reversible signal changes following the increase in concentration, distinct emission potentials, and minimal interluminophore cross-talk. Since CdS quantum dots (QDs)/Tris-(bipyridine)-Ru (II) (Ru(bpy)_3_^2+^), the first donor and receptor pair, was developed, many novel potential-resolved RET pairs with unique properties have been exploited for ratiometric ECL sensors 42 (Table 1). For example, Han *et al.* 43 developed novel dual-ECL luminescent Janus particles (JPs), which are asymmetric heterostructures comprising Ru (II) complexes and luminol polymers. They enable the simultaneous generation of two strong ECL emissions while eliminating potential signal interference, reducing background interference, and improving ECL intensity. An ultrasensitive dual-quenching Janus ECL-RET strategy using Ru-Lu JPs and dyes (FAM and cyanine dye (Cy5) as donors-acceptors combined with catalytic hairpin assembly amplification was proposed for the simultaneous determination of dual-miRNAs. Similarly, Li *et al.* 44 developed a dual-potential ECL platform based on the RET system between SnS_2_ QD hybrid Eu metal-organic frameworks (MOFs) as the cathodic emitter and luminol-capped Pt-tipped Au bimetallic nanorods as the anodic luminophor for ultrasensitive KAN detection.

**Figure 1:**
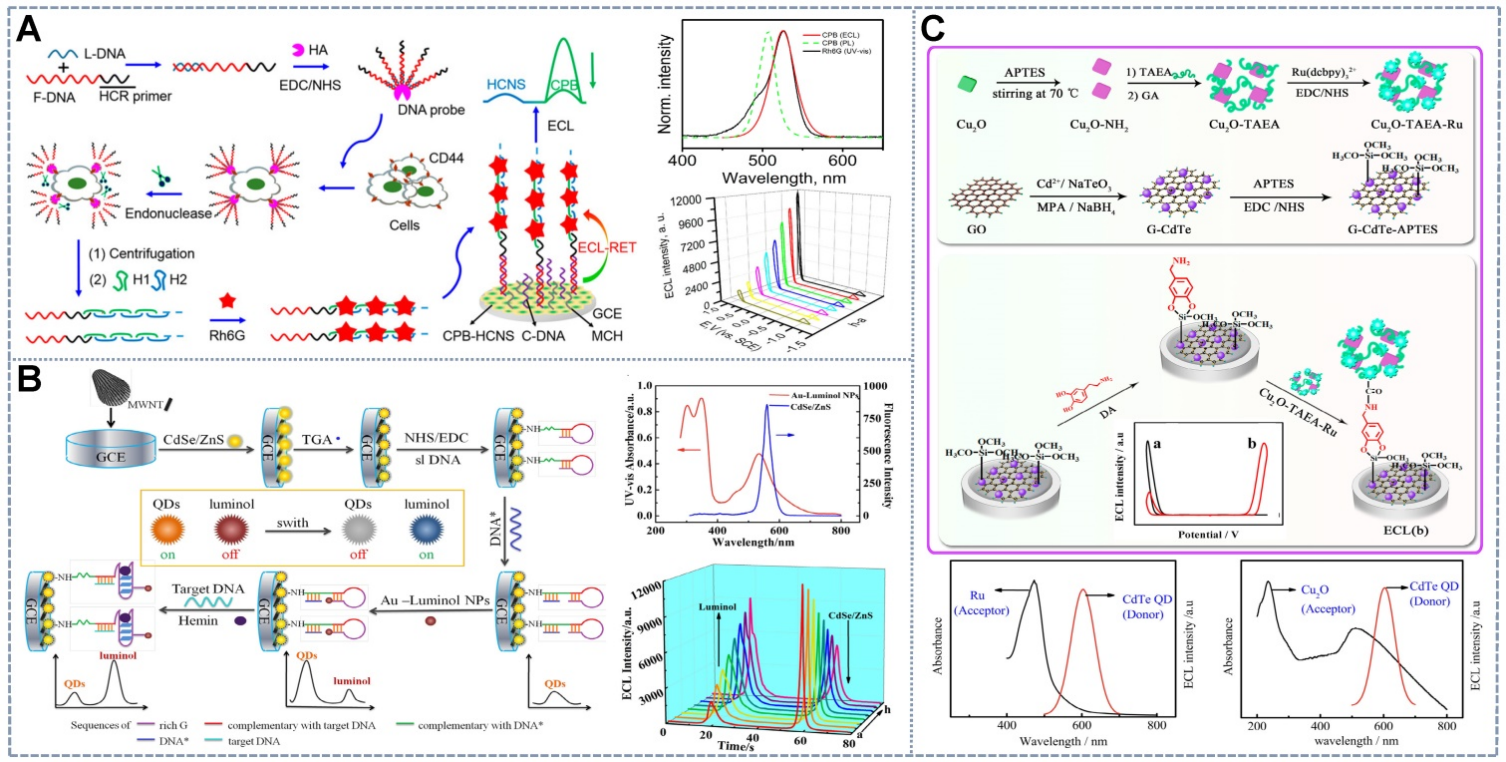
Schematic of the potential resolved ECL-RET biosensors based on **(A)** the ECL-RET system with luminophores with high color purity and facile band controllability. Adapted with permission from 40, copyright 2020 American Chemical Society; **(B)** universal enhancer or quencher. Adapted with permission from 51, copyright 2018 Elsevier B.V.; and **(C)** signal regulation materials. Adapted with permission from 55, copyright 2016 Elsevier B.V.

However, some ECL-RET systems cannot realize ideal energy transfer between luminophores 45. So, some materials that help control ECL emissions, like catalytic (hemin/G-quadruplex DNAzyme 34, horseradish peroxidase (HRP)/AuNRs 36), carriers (poly(amidoamine) (PAMAM), TiO2 mesocrystals, helical carbon nanotube (HCNTs) 46) or quencher (GO 23, Cy5 39) are introduced into ratiometric systems. For example, Hao *et al.* 47 introduced Au-luminol and CdS QDs as signal probes in an aptasensor. However, the deficient RET properties of the aptasensor hindered its application in ratiometric systems. Therefore, the quencher fluorophore Cy5 was combined with CdS QDs to perform ECL-RET with luminol and amplify the CdS QD signal. These effects enabled the self-tuning of the two emissions. Similarly, as shown in Figure 1A, Cao *et al.* 40 proposed CsPbBr_3_ nanocrystal (CPB), which acted as an ECL emitter with high color purity (with a full-width-at-half-maximum (FWHM) of 12-25 nm) and facile band tunability through halogen exchange. These characteristics make CPB a perfect ECL donor and facilitate the search for available ECL receptors. Then, the all-inorganic perovskite anodic emitter CPB was *in situ* assembled into hollow graphitic carbon nitride nanospheres (HCNSs) for a matching band-edge arrangement to enhance its ECL performance. The HCNSs also served as the cathodic emitters for ratiometric analysis. Given that the emitter complex lacked signal regulation ability, rhodamine 6G (Rh6G) was introduced into the system. Rh6G can be loaded onto the long DNA duplexes generated through the hybridization chain reaction triggered by the addition of the detector and subsequently dramatically quench the anodic ECL of CPB. Therefore, the signal from CPB indicated the analyte concentration and that from HCNS was used as an internal standard to calibrate the signal. However, the application of RET-based ECL ratiometric sensors is limited by the lack of energy-tunable materials, especially ECL-RET donors and acceptors that perfectly overlap.

Researchers have introduced a dual-role energy transfer intermediate that integrates the quenching effect and catalytic properties to address the lack of ECL-RET luminophore pairs. Metal nanoparticles (NPs), such as platinum NPs 47 and gold NPs (AuNPs) 34 are the most commonly selected energy transducers because of their different effects on the ECL of CdS and luminol. The most common pattern of the intermediate ECL-RET strategy is constituted by luminol-functionalized AuNPs and CdS-C nanoflowers (NFs) 48/G-CdTe QDs 42/CdTe@CdS QDs 23. The mediatory function of AuNPs comprises (a) the anodic ECL enhancement attributed to the increased active electrode area and the catalytic effect on luminol oxidation 48 and (b) the cathodic quenching resulting in nonradiative energy dispersion or Förster RET from QDs 49. Additionally, at a certain distance, excited-state QDs could stimulate the surface plasmon resonance of AuNPs, which generates powerful local electric fields and then leads to additional excited-state QDs 50 to enhance the intensity of cathodic ECL. The formation pattern of the dual-role energy transfer intermediate in the ratiometric platform is shown in Figure 1B, in which the binding of target molecules changes the conformation of rigid structures of the DNA chain, thus altering the distance between CdSe/ZnS and AuNPs-luminol such that two ECL signals at different potentials are changed oppositely to form the ratiometric ECL biosensor 51. At present, many RET-ECL ratiometric systems have been developed on the basis of dual-role intermediates, which include luminol-Str-AuNPs/CdS 52, Au-luminol/CdSe/ZnS QDs 51, luminol-AuNPs/CdS-C NFs 48, luminol/CdTe/CdS/ZnS QDs 39, luminol/CdTe@CdS QDs 34, Ag-PAMAM-luminol NCs/graphitic carbon nitride (g-C_3_N_4_) 53, luminol/GQD 24, luminol/CdS NCs 5, and CdTe/CdS/ZnS QDs-HRP/AuNRs-luminol 39.

Nevertheless, the above ECL-RET strategies lack universal applicability because of the demanding requirement for sufficient spectrum overlap between the ECL energy donor and acceptor. Therefore, a universal enhancer or quencher is desirable for dual-potential ratiometric ECL sensors. Wang *et al.* 54 proposed a superior and universal quencher, cuprous oxide (Cu_2_O) (Figure 1C). Cu_2_O, a p-type semiconductor, has been confirmed to consume dissolved oxygen by catalyzing oxygen reduction during the negative scan and thus could be an effective quencher for luminophores with dissolved O_2_ as the co-reactant. Similarly, Fu *et al.* 55 utilized Cu_2_O to form a self-enhanced Cu_2_O-TAEA-Ru (II) complex as an anodic ECL label and embellished graphene-CdTe QDs as the cathodic ECL probes on the surface of the electrode to develop a dual-potential ECL ratiometric sensor for the sensitive detection of dopamine. The ratiometric detection of dopamine was realized via the ECL-RET from G-CdTe QDs to TAEA-Ru, as well as the dual quenching effects of Cu_2_O on G-CdTe QDs, namely, the ECL-RET from G-CdTe QDs to Cu_2_O and the consumption of the co-reactant O_2_ by Cu_2_O. Other intermediates have also been reported by Zheng *et al.* 56, who first utilized ferrocenecarboxylic acid (FCA) as a bifunctional reagent to regulate the ratiometric ECL sensing mode by virtue of the double effects of FCA in quenching the ECL of Ru (dcbpy)_3_^2+^ through inhibiting the generation of excited-state Tris (2,2'-bipyridine)Ru (II) (Ru (bpy)_3_^2+*^) and enhancing luminous efficiency by catalyzing the generation of the reactive oxygen species (ROS, OH· and O_2_^·-^) that facilitate the oxidation of luminol.

Above all, the ECL-RET strategy is gaining popularity as a potent signal regulation approach in ratiometric ECL systems. Several energy transfer intermediate reagents, including metal NPs, Cu_2_O, and FCA, have been widely used. Additional RET luminous pairings with potential resolution qualities will most likely be constructed in the future. Also, finding RET intermediate compounds is essential for solving the lack of effective pairs of energy donors and acceptors and for increasing the number of ECL-emitting systems used in potential-resolved ratiometric systems.

### Competition Strategy

In addition to ECL-RET-based potential-resolved systems for establishing ratiometric sensors, systems based on the competitive strategy have been widely constructed given their inherent superiority in triggering the reverse signal changes of dual potential-resolved ECL emitters 22. The competitive strategy of co-reactant consumption has been extensively investigated and maturely developed, and more recently, several research contends for the binding space of the electrode surface, known as steric hindrances competition systems, have emerged to form the ratiometric system.

**Figure 2:**
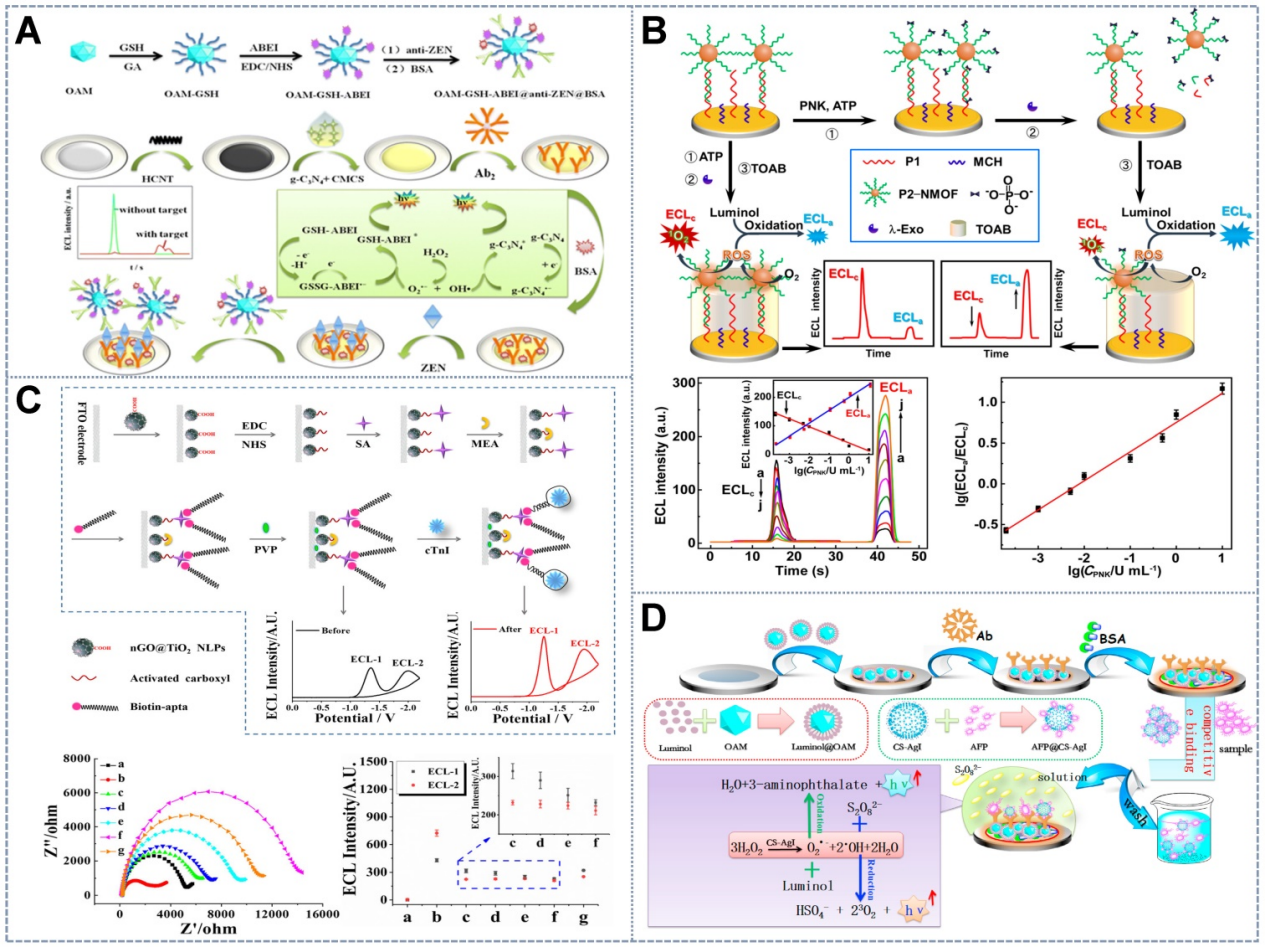
Schematic of potential-resolved competition-strategy sensors with **(A)** H_2_O_2_ as the co-reactant. Adapted with permission from 62, copyright 2020 Springer; and **(B)** dissolved O_2_ as the co-reactant. Adapted with permission from 65, copyright 2020 American Chemical Society. **(C)** Steric hindrance strategy. Adapted with permission from 69, copyright 2019 American Chemical Society. **(D)** Immune competition mechanism. Adapted with permission from 71, copyright 2016 Elsevier B.V.

By relying on the different catalytic efficiencies of luminophores for their shared co-reactants, the consumption of competitive co-reactants between a pair of potential-resolved luminophores was utilized for ratiometric ECL sensors 41. Various competitive co-reactant-consuming ECL ratiometric systems have been constructed on the basis of H_2_O_2_ because of its combined reducing and oxidizing properties. For example, Fang *et al.* 62 used the self-enhanced glutathione (GSH)-*N*- (aminobutyl)-*N*- (ethylisoluminol) (ABEI) composite as an anodic probe and labeled the anti-antibody with the cathodic probe g-C_3_N_4_ nanosheets (NSs) and horseradish peroxidase (HRP) in a zearalenone (ZEN) immunoassay. When immune recognition occurred, the anodic ECL signal increased and the cathodic ECL signal decreased due to the competitive consumption of the co-reactant H_2_O_2_ by the g-C_3_N_4_ NSs and GSH-ABEI (Figure 2A). Furthermore, peroxidase accelerated the decomposition of H_2_O_2_ into ROS, thus simultaneously amplifying the blue ECL of ABEI and the green ECL of g-C_3_N_4_ and contributing to the widening of the linear range and the increase in sensitivity 63. Other luminophore pairs with H_2_O_2_ as the shared co-reactant, such as LuAuNPs/AuNPs@CNNS 64 and CdS QDs/luminol 33, have been utilized in this ratiometric ECL sensing system. However, several inherent defects in H_2_O_2_ impede its application in the competitive consumption strategy: (1) H_2_O_2_ has been identified to be an unstable co-reactant because it is prone to decomposition into OH· radicals at ambient temperature. Furthermore, when present in excess, H_2_O_2_ molecules may annihilate each other. This phenomenon greatly affects the stability and reproducibility of bioanalysis 65. (2) H_2_O_2_, an exogenous co-reactant, has a relatively low catalytic efficiency that leads to its higher consumption of reagents than the endogenous co-reactants. (3) Given its biological toxicity and volatility, H_2_O_2_ poses certain safety risks, which will also affect measurement deviation 63. So, it is very important and could be very useful to look for a stable and green co-reactant for the competitive consumption strategy in ratiometric ECL systems.

Compared with H_2_O_2_, dissolved O_2_ in aqueous solutions is a more promising endogenous co-reactant candidate, especially in luminol ECL systems, due to its higher stability and nontoxicity. Dissolved O_2_ in the solution can be used directly as a co-reactant or can be continuously adsorbed and dissociated at the catalytic interface during the oxygen reduction reaction (ORR) to generate ROS or H_2_O_2_ for subsequent ECL reactions. Zhang *et al.* 65 proposed a dissolved O_2_-competing ECL catalytic interaction involving two-dimensional copper-based zinc porphyrinic MOF (CuTCPP (Zn))NSs and luminol. The constructed GNPs would diminish the cathodic ECL of the singlet oxygen formed through the electrocatalytic reaction of the two-dimensional CuTCPP (Zn) due to RET, while the GNPs with strong electrocatalytic activity would boost the anodic emission of luminol (Figure 2B). The ECL process driven by the competitive mechanism had a lower detection limit and a broader linear relationship in the detection of polynucleotide kinase (PNK) than single-signal-driven ECL sensors due to the participation of dissolved O_2_. The same dissolved O_2_ competing system with the anodic emitter ABEI, the cathodic emitter CdTe QDs 66, and 2-(dibutylamino) ethanol (DBAE)/lucigenin 67 demonstrated excellent stability, selectivity, and reproducibility. The co-reactant competitive ratiometric ECL mechanism based on dissolved O_2_ or its products may provide a new pathway for further research on green and highly sensitive ECL biosensing systems.

The steric hindrance competition strategy has been introduced into ratiometric ECL systems with multiple recognition modes in an effort to form ultrasensitive and economical target-mediated self-calibrated ECL sensors. For example, on the basis of a molecularly imprinted (MIP) recognition platform, Cao *et al.* 68 constructed a simple and universal steric hindrance ratiometric ECL sensor with TiO_2_-Ru(bpy)_3_^2+^NPs as the anodic luminophore and PEI-CdS QDs as the cathodic luminophore and anodic co-reactant, thus avoiding the addition of multiple co-reactants. The SA-imprinted poly [3-aminophenylboronic acid] film was prepared on the electrode as the recognition element and then modified with two luminophores, which strictly hindered electron transfer and impeded the direct contact of the cathodic co-reactant H_2_O_2_ with PEI-CdS. After the binding, elution, and re-recognition of SA on the MIP, the sensor exhibited an obvious increase in the intensity of the ECL signal from Ru(bpy)_3_^2+^ and an evident decline in the intensity of the ECL signal from PEI-CdS because SA could assist the ECL reaction of Ru(bp)_3_^2+^ by acting as an anodic co-reactant and the cathodic reaction was impeded by the occupation of the imprinted cavities. Furthermore, Han *et al.* 69 applied the steric hindrance strategy based on a label-free ECL aptasensor for the determination of cardiac troponin I(cTnl) (Figure 2C). In this sensor, the dual-signal nanoluminophore nanographene oxide (nGO)-wrapping titanium dioxide (nGO@TiO_2_NLPs) that was fabricated on the ECL interface generated two potential-separated ECL. Aptamers for cTnI assembled on nGO@TiO_2_NLP for target capture. After binding with cTnI, the aptamer becomes too rigid to cling to the surface of the electrode, thus leaving space for charge transfer. This effect resulted in the ratio enhancement of the two ECL signals of nGO@TiO_2_NLPs. Interestingly, Wang *et al.* 70 introduced a catalyzed hairpin assembly-driven bipedal DNA walker into the ratiometric strategy to form the Au@luminol-HP1/Au@CDs-HP2 duplex on the sensing interface. After the introduction of Au@CDs-HP2 into the electrode-bound Au@luminol-HP1 hairpins, a cathodic ECL signal was observed as a result of the addition of Au@CDs, and the anodic ECL signal decreased because steric hindrance from the Au@CDs-HP2 complex blocked the interaction between Au@luminol and H_2_O_2_. By exploiting the high luminescence and excellent biocompatibility of AuNPs/luminol and AuNPs/carbon dot hybrids, the proposed system exhibited superb ratiometric performance and also offered a reliable method for early clinical diagnostics and cutting-edge biomedical research.

The target-involved competitive immunoassay, another competitive ratiometric system, is a promising sensing protocol because of its minimalist design and ultrasensitive determination. Zhang *et al.* 71 constructed an enzyme-labeling competitive immunoassay for determining alpha fetoprotein(AFP) The immunorecognition interface was constructed by incubating the mixture solution of chitosan-functionalized silver iodide (CS-AgI)-labeled AFP (CS-AgI@AFP) and the target AFP on the antibody (Figure 2D). With the increase in the concentration of AFP, the amount of the CS-AgI@AFP immobilized on the electrode decreased due to the competitive recognition of AFP, resulting in the reduced catalytic effect of CS-AgI for the decomposition of H_2_O_2_. This effect consequently amplified the potential-resolved ECL of luminol and S_2_O_8_^2-^ in the ratiometric ECL strategy. The proposed competitive immunoassay omitted the introduction of secondary antibodies, thus facilitating electrode transfer on the recognition platform and guaranteeing its outstanding sensitivity over the common sandwich immunoassay.

### Enzyme-based Ratiometric Strategy

**Figure 3:**
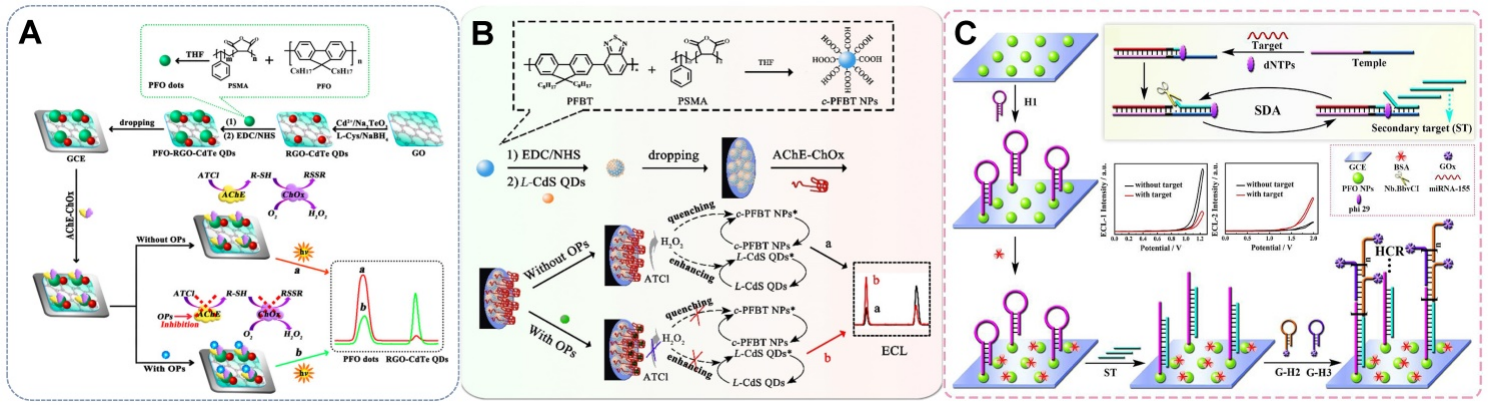
Schematic of the potential-resolve enzymatic strategy sensors based on the reverse variation of the substrate O_2_ and H_2_O_2_ in **(A)** the AChE-ChOx enzyme system. Adapted with permission from 75, copyright 2017 American Chemical Society; single H_2_O_2_ substrate as the bifunctional moderator in **(B)** the glucose oxidase system. Adapted with permission from 74, copyright 2020 Elsevier B.V.; and **(C)** the AChE system. Adapted with permission from 81. Copyright 2021 American Chemical Society.

The potential-resolved enzyme-based ratiometric strategy is an enzyme-catalyzed ratiometric method. It exploits the enzymatic reactions between the reactant and product, which act as the co-reactants of two different ECL emitters, to attain the distinguishable changes in the potentials of inverted ECL signals for ratiometric determination. This platform possesses excellent accuracy, reliability, and sensitivity and is therefore an alternative for alleviating the shortage of suitable luminous pairs, the limitation of the distance dependence of RET, and the cross-talks between co-reactants in luminating systems 74. Various types of enzymes, especially oxidoreductases, such as the acetylcholinesterase (AChE)-ChOx enzyme system and xanthine oxidase (XOD), have been utilized to catalyze the production and consumption of ECL signal regulators, including O_2_ and H_2_O_2_, to realize signal conversion. Chen *et al.* 75 integrated two desirable ECL emitters, namely, polymer poly(9,9-dioctylfluorene) (PFO) dots and CdTe QDs, that feature ideal properties, such as potential-resolved ECL signal emission and freedom from mutual energy transfer. The PFO dots and CdTe QDs took the enzymatic reaction reactant dissolved O_2_ and product H_2_O_2_ as their exclusive co-reactants, respectively (Figure 3A). Such a construction strategy overcomes the limitations of using exogenous co-reactants and the discommodity of oxidase-based determination in the traditional ECL ratiometric method. However, given that the luminous efficiency of this system suffered from the poor water solubility of PFO, the same group established another anodic luminophor luminol with favorable water solubility by using H_2_O_2_ as the co-reactant for the determination of the XOD substrate hypoxanthine (Hx) 76. In the presence of Hx, XOD catalyzed oxidation, resulting in the *in situ* consumption and generation of dissolved O_2_ and H_2_O_2_, which served as the co-reactants of rGO-CdTe QDs and luminol, respectively. This phenomenon resulted in the reduction of the cathodic signal and the enhancement of the anodic signal, upon which Hx was ratiometrically detected with a low detection limit. The *in situ* consumption and generation of co-reactants via enzyme catalysis not only offered an ingenious tactic for the determination of substrate concentration but also alleviated the error introduced by exogenous co-reactants in common ratiometric systems.

Other commonly constructed enzyme ratiometric sensors are based on single H_2_O_2_. H_2_O_2_ could serve as a bifunctional moderator to mediate the opposite change of the two signals because it is taken as a substrate or product in various enzymatic reactions 77-79. For example, Zhang *et al.* 80 designed metal-organic gel catalyst Fe(III)-organic gel matrices for the efficient enhancement of the ECL signals of luminol and CdS QDs through the conversion of the co-reactant H_2_O_2_ into ROS via an electrochemically mediated Fenton-like reaction. A dual-potential ratiometric strategy was proposed for the accurate cytosensing and direct evaluation of VEGF_165_ subtypes on cell surfaces. Taking advantage of the quenching and enhancing adverse regulatory effect of H_2_O_2_ produced by GOx, Liu *et al.* 74 achieved the ratiometric sensing of miRNA-155 on the basis of the ratio of two ECL signals emitted by PFO NPs at two potentials (+1.25 and +1.95 V) (Figure 3B). Similarly, He *et al.* 81 developed a bifunctional moderator-powered enzymatic biosensor based on a dual-signal combined nanoprobe and the enzyme AChE. In this system, AchE catalyzed the substrate acetylthiocholine to produce H_2_O_2_, which quenched the anodic ECL signal from c-PFBT NPs and promoted the emission of the cathodic ECL signal from L-CdS QDs for organophosphorus analysis (Figure 3C). Taking into account the problems with RET and exogenous co-reactants, the above-mentioned enzymatic biosensors avoided the troublesome step-by-step assembly of two signal probes in ECL sensor and also provided a new measure for facilitating the opposite changes of two signals.

The enzyme-labeling amplification strategy has been proven to intensify ECL signals in ratiometric ECL protocols to improve the sensitivity of ECL detection. Fang *et al.* 63 labeled anti-ZEN with HRP, which accelerated the decomposition of H_2_O_2_ into ROS, thus significantly amplifying the potential-resolved ECL signal of ABEI and GSH. This effect facilitated sensitive and reliable ZEN analysis with a wide linear range in corn hazelnut samples. Nevertheless, the generalization of the enzyme-labeling strategy is impeded by some intrinsic drawbacks, such as expensive preparation and purification processes, environmental susceptibility, and undesirable stability. Artificial enzyme mimics have received widespread attention because of their alternative catalytic activities for signal amplification and high stability, reasonable cost, spatial structure flexibility, and storage stability. Zhang *et al.* 71 utilized the biomimetic catalyst CS-AgI to catalyze the decomposition of H_2_O_2_ into O^2·-^ and ·OH, which served as the co-reactants of the potential-resolved emitters luminol and K_2_S_2_O_8_, respectively. This approach resulted in the enhancement of dual ECL responses. The enzyme-triggered amplification protocol provides a promising strategy for improving the sensitivity of potential-resolved dual-electrode reaction systems, and its application in visualized detection has a bright future.

### Single Luminophore Ratiometric ECL Strategy

Although the reported conventional ratiometric ECL systems based on ECL-RET, competitive strategy, or enzymatic reactions have provided more reliable and sensitive sensing signals than those based on traditional single emitters, they mainly require two elaborately selected potential-resolved luminophores and complex combinations of co-reactants, as well as multiple assembly steps and labeling courses. Existing studies have found that many single luminophores can produce dual ECL signals under the application of different potentials and in the presence of corresponding co-reactants 83. Through the reasonable design of the sensing interface, these luminescence systems could considerably expand the ratiometric ECL strategy.

**Figure 4:**
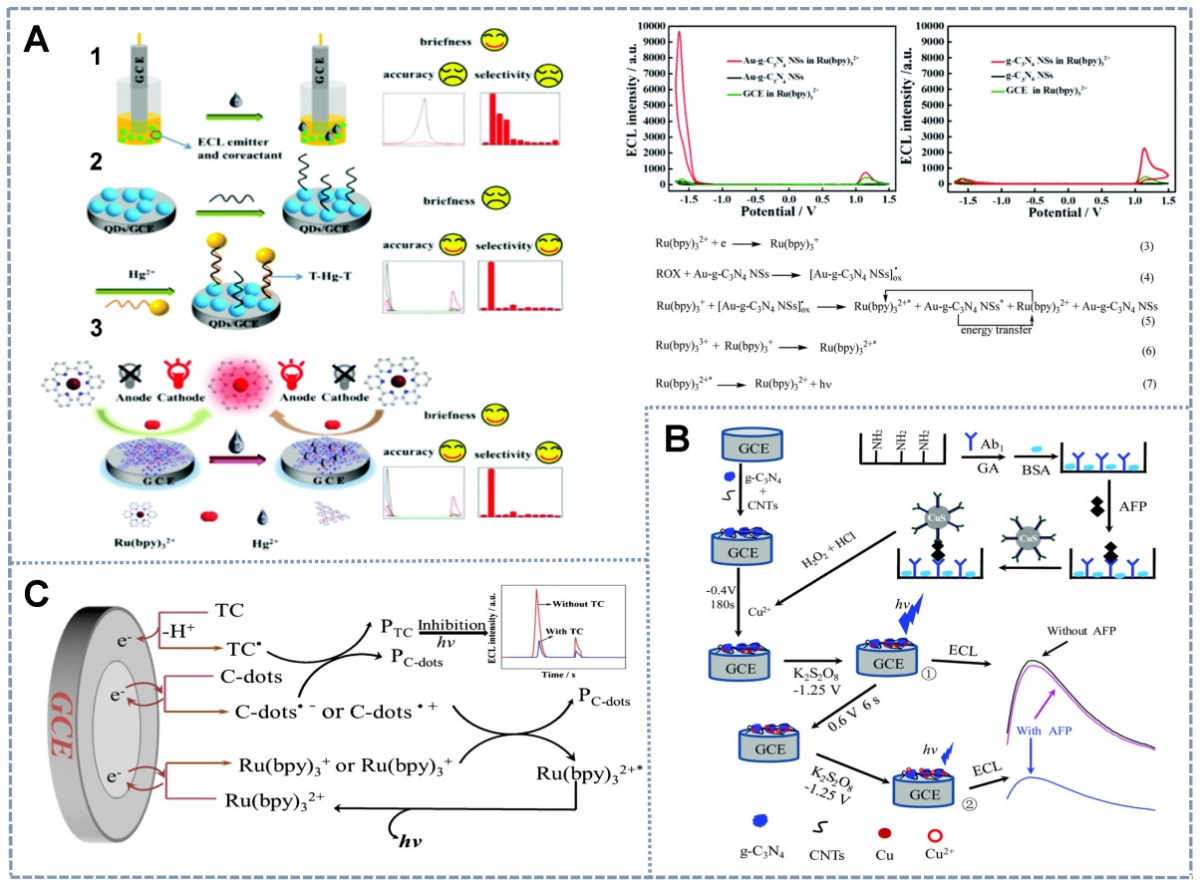
Schematic of single luminophore ratiometric ECL sensors based on dual co-reactants. **(A)** G-C_3_N_4_ and Au-g-C_3_N_4_ as the co-reactants of Ru(bpy)_3_^2+^ for Hg^2+^ detection. Adapted with permission from 84, copyright 2020 Royal Chemical Society. **(B)** K_2_S_2_O_8_ and TEA as the co-reactants of porous g-C_3_N_4_ NSs for AFP detection. Adapted with permission from 53, copyright 2020 Royal Chemical Society. Single-luminophore ratiometric ECL sensors based on **(C)** C-dots as the single co-reactant of Ru(bpy)_3_^2+^ for the determination of the antibiotic TC. Adapted with permission from 87, copyright 2019 Springer.

The special properties of the electrochemical reaction between the analyte and the dual-signal single luminophore are mainly utilized via an ingenious design to realize the construction of ratiometric determination systems. Due to their high quantum efficiency and anodic or cathodic ECL emission properties upon reaction with different co-reactants, Ru(bpy)_3_^2+^ and its derivatives are the most widely used ECL emitters in single luminophore-based ratiometric systems. However, the lack of modifiable cathodic co-reactants impedes their application. Cao *et al.* 84 proposed the novel cathodic co-reactant Au-g-C_3_N_4_ NSs of Ru(bpy)_3_^2+^ (Figure 4A) prepared via the in-situ synthesis of AuNPs on the surface of g-C_3_N_4_ NSs, which is the anodic co-reactant of Ru(bpy)_3_^2+^. They utilized the formation of gold amalgam between Hg^2+^ and Au to develop a label-free Hg^2+^ ratiometric sensor by inhibiting the activity of AuNPs. This effect simultaneously hindered cathodic ECL emission and enhanced anodic emission. Furthermore, Chen *et al.* 85 synthesized the single-emitter nitrogen-doped graphene QDs (NGQDs) by using the oxygen electrolytic products O_2_^·-^ and HO_2_^-^ under positive and negative potentials as the anodic and cathodic co-reactants, respectively, to produce bipotential responses. Then, the Co^2+^ ion was determined on the basis of the amplification of the anodic signal by the catalytic action of Co^2+^ during the intermediate steps of the anodic ECL process and the quenching of the cathodic ECL intensity due to the Co^2+^ elimination effect of the excited state (NGQDs^*^) generation. In addition, Shang *et al.* 86 reported that dual-ECL signals could be generated by distinct ECL reactions triggered by GPPCN NSs at anodic and cathodic potentials to distinguish trace amounts of target metal ions when the concentrations of interfering metal ions were several times higher. At different driving potentials, different metal ions showed variable signal quenching and enhancement likely due to the diversity of the energy level matching between metal ions and GPPCN NSs and the catalytic interactions of the intermediate species in ECL reactions. As a result, the accuracy and reliability of sensors based on the ECL of GPPCN NSs for metal ion detection were considerably enhanced, the need for any labeling or masking reagents was eliminated, and the production of false-positive results by interferential metal ions was effectively prevented. Therefore, using the special chemical reactions between different metals and ECL materials, it is possible to measure the amount of metal ions with high sensitivity and selectivity from the ratio of two ECL signals at different potentials.

The proposed strategy for the construction of sensing interfaces based on signal emitters could be utilized not only for the detection of metal ions but also for the determination of large molecules, such as proteins, aptamers, and even cells with complex designs. Labels and substrates with potential discrimination properties without the chemical characteristics of metal elements are needed for the detection of biomaterials. Chen *et al.* 53 reported a biometallization signal amplification strategy. In this strategy, porous g-C_3_N_4_ NSs were used as the single luminophore with K_2_S_2_O_8_ and triethanolamine (TEA) as the cathode and anode co-reactants (Figure 4B), and CuS NPs were applied as the tag to build an ultrasensitive immunoassay for AFP detection. Specifically, the sensing interface consisted of g-C_3_N_4_ NS-modified carbon nanotubes (CNTs) as the electrode base and second antibodies labeled by CuS NPs as the probe. The ECL determination process was coupled with the anodic stripping voltammetry strategy to amplify the ECL signals of the electrochemical deposition of Cu from the CuS NP tag at a negative potential and the dissolution and enrichment of Cu^2+^ within the Helmholtz layer of GCE/CNTs-g-C_3_N_4_ at a positive potential that significantly quenched the g-C_3_N_4_ emission. The cathodic signal was taken as the internal reference, and the anodic signal that changed reversibly with AFP was quantified by using a ratiometric ECL system. The combination of ratiometric ECL with electrochemical enrichment and biometallization is a useful strategy for enhancing the sensitivity and reproducibility of immunoanalysis.

Another strategy relies only on a single emitter and a single co-reactant to attain the simplest ratiometric ECL system. In this system, the ratio of the dual signal with a single-directional change at separate potentials was used as the output signal. For the detection of the antibiotic tetracycline (TC), Hu *et al.* 87 employed Ru(bpy)_3_^2+^ as the single electrochemical probe that exhibited a double ECL response with potential scanning from -3.5 V to +2 V and C-dots as the co-reactant (Figure 4C). The dual signals of Ru(bpy)_3_^2+^ decreased with the increase in TC concentration due to the inhibitory effect of C-dots on TC during the electrochemical reaction. Similarly, Han *et al.* 69 reported the new core-shell-like structured nanoluminophore nGO@TiO_2_NLPs. The nGO@TiO_2_NLPs were synthesized through a one-pot hydrothermal method and exhibited potential-resolved property with the co-reactant K_2_S_2_O_8_ at pH = 7.0. The peak potentials of ECL-1 and ECL-2 were observed at -1.27 and -1.85 V and were emitted by the TiO_2_ and nGO moieties of the nGO@TiO_2_ NLPs, respectively. After the addition of the target cTnI, the dual signal was simultaneously quenched due to the steric hindrance of the aptasensor. On the basis of the ratio of ECL-1 and ECL-2 intensities, a label-free ratiometric ECL system was constructed for the detection of the AMI biomarker cTnI. The system enabled the superior, accurate, sensitive, specific, and rapid early detection of AMI. Therefore, the exploitation of the dual-signal strategy based on one luminophore has a bright future given its economy, environmental friendliness, and simple system construction.

### Internal Standard Ratiometric Sensing Strategy

The internal standard ratiometric sensing strategy is another protocol for constructing a ratiometric signal-based sensing platform in conjunction with the dual-potential based strategy mentioned above. While in the internal standard ratiometry scheme, exploiting the ECL luminophore pair to generate differentiable ECL working signal and internal reference in one single scan is a key factor. Thus the potential-resolved ECL system is the foundation stone of internal reference ratiometric sensors 88.

**Figure 5:**
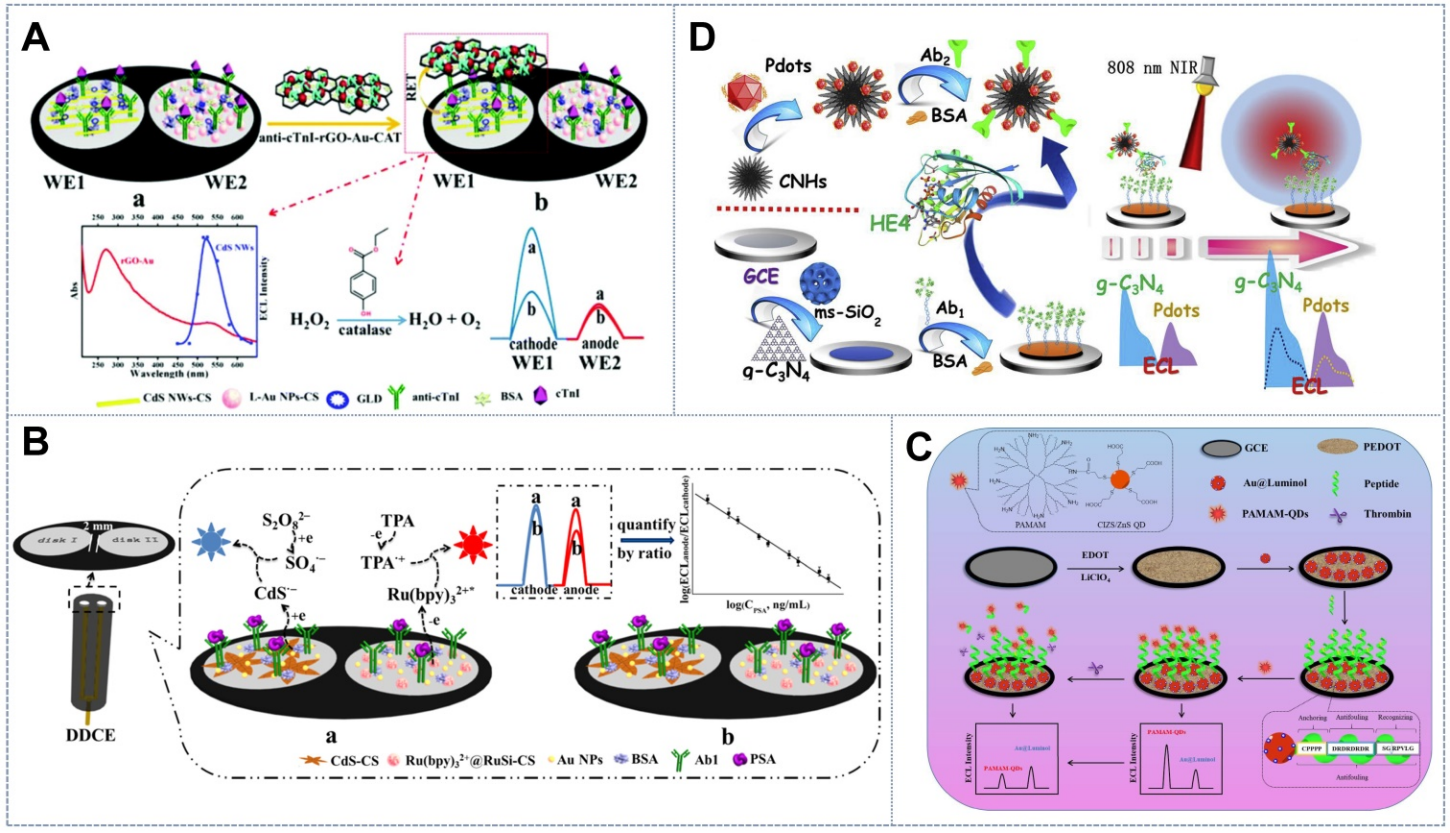
Schematic of the internal standard ratiometric ECL sensor with the physically separated double disk electrode (WE1 and WE2) in **(A)** a dual-disk inner reference ratiometry system. Adapted with permission from 90, copyright 2020 Royal Chemical Society; **(B)** a DDCE label-free system. Adapted with permission from 88, copyright 2017 Elsevier B.V. Schematic of the internal standard ratiometric ECL sensor with the physically separated double disk electrode (WE1 and WE2) with single electrode assembled with an internal reference signal probe and working signal probe in **(C)** a ratiometric antifouling ECL biosensor based on PAMAM-CIZS/ZnS QDs. Adapted with permission from 91, copyright 2020 Elsevier B.V.; and **(D)** with photothermal amplification strategies. Adapted with permission from 32, copyright 2019 Elsevier B.V.

The internal standard ratiometric strategy was initially realized on the basis of physically separated double disk electrodes (WE1 and WE2). The mechanism of the internal reference scheme on spatial-resolved disks is based on the self-calibration of the ratio of ECL signals from WE1 (as the working signal) to WE2 (as the internal reference standard signal). Feng *et al.* 89 fabricated an internal standard ratiometric aptasensor on a homemade screen-printed carbon electrode (SPCE) for the detection of the antibiotic chloramphenicol (CAP) for the first time. The working signals from the anodic luminophore Lu-AuNPs were compared with the internal standard signals from CdS QDs at negative potential to reduce environmental disturbance. Cao *et al.* 90 presented a novel dual-disk inner reference ratiometry method for cTnI analysis (Figure 5A). WE1 and WE2, which were considered as the working and reference electrodes, respectively, were modified with CdS nanowires (CdS NWs) as the potential-resolved cathodic emitter and luminol-AuNPs (L-AuNPs) as the anodic emitter. Dual-signal-amplifying probe anti-cTnI-reduced grapheme oxide-AuNPs-catalase loaded onto the WE1 functioned as the RET acceptor of CdS NWs and as the CAT catalyzing the co-reactant (i.e., H_2_O_2_) consumption that resulted in a prominent decrement in ECL along with an increase in cTnI concentration. Internal and external interferences were remarkably calibrated by the internal reference signal from WE2 loaded with L-AuNPs embedded with a fixed amount of cTnI. Furthermore, the physical separation of the workspaces of different emitting systems conspicuously mitigated the potential cross-talk and cross-reactivity between luminophores, thus effectively increasing the specificity and reliability of ECL performance. Wang *et al.* 88 designed a label-free spatial-resolved dual-signal-output ratiometric biosensor based on a dual-disk glassy carbon electrode (DDCE) for the prostate-specific antigen (PSA) assay (Figure 5B). Self-synthesized three-dimensional flower-like CdS assemblies were used as the cathodic ECL emitters. Ru(bpy)_3_^2+^@RuSi NPs, which acted as the anodic ECL emitter, emitted the working signal that served as the reference signal to alleviate environmental interference during biological recognition. The exact same building process and unified building pattern between the two disks universally endowed the dual-disk electrode-based internal standard ratiometric strategy with an ultra-accurate and reliable biosensing scheme.

Further studies assembled the internal reference signal and working signal in one electrode as a concise construction mode to generalize the internal standard ratiometric sensing strategy to a common single-disk electrode. The ratio of dual potential-resolved signals could be acquired for target concentration quantification in the single-sensing interface by modifying one emitter to act as the electrode substrate and internal reference and modifying another potentially distinguishable emitter on the bioprobe as the working signal tag. The favorable accuracy, reliability, and sensitivity of the bioassay were thus realized. Xu *et al.* 91 constructed a ratiometric antifouling ECL biosensor based on the novel benign water-soluble self-enhanced luminescence probe PAMAM-CuInZnS/ZnS QDs (PAMAM-CIZS/ZnS QDs) (Figure 5C). The electrode was modified with the anodic ECL emitter Au@Luminol as the internal standard molecule. The noble metal AuNPs could also simultaneously improve the conductivity of the electrode. Ding *et al.* 92 designed a bimodal ECL system based on CdTe QDs and luminol as the potential-resolved luminophores. CdTe QDs were tagged onto aptamers for cancer cell capture, while luminol molecules, which served as the internal standards, were entrapped in the conducting polymer hydrogel on the electrode surface. In further research, Fang *et al.* 32 introduced photothermal amplification strategies into the internal reference ratiometric ECL system to enhance ECL signals. To do so, the cathodic ECL emitter complex gC_3_N_4_@ms-iO_2_ was designated as the sensing platform and the anodic ECL emitter complex polymer dots (Pdots)@CNH was designated as the photothermal probe (Figure 5D). In this approach, CNHs were used as the thermal conversion unit to increase the electrode surface temperature given their exceptional photothermal property at 808 nm. This effect can convert laser energy into heat for temperature elevation, thus further amplifying the ECL signal. Above all, through incorporation with the internal standard strategy, the developed potential-resolved based ratiometric ECL sensor exhibited satisfactory accuracy, reliability, and practicality in complex biological media. These properties verified its promising utilization in home healthcare and early clinical diagnosis.

## Potential-resolved Strategy in Multiplex ECL Analysis

The simultaneous detection of multiple markers in the same sample requires the detection of multiple targets in a single run or in the same system. This approach has more advantages, such as smaller sample volume, lower cost, higher analysis throughput, and the ability to acquire more information from one sample, than single-target detection 95,96. Furthermore, the detection of multiple biomarkers is vital for improving the sensitivity and accuracy of cancer diagnosis because tumor markers and tumors lack one-to-one correspondence but instead present complex correlations and stage-dependent differences 97,98. Therefore, the detection of multiple biomarkers has received increased attention, and additional novel designs have been rapidly developed.

In the potential-resolved strategy, ECL probe signals correspond to each onset working potential. Therefore, the concentration of multiple biomarkers can be revealed through only a simple potential scan of the same sensing interface when it is labeled with different signal probes. Such an approach is timely and convenient. This detection method is called potential-resolved multiplex ECL detection 99. In contrast to the ratiometric potential-resolved system, some conventional ECL systems are inapplicable to multiplex ECL detection. In these systems, ECL signals are produced by one luminophore with different co-reactants or with a single co-reactant in different chemical states 87. Therefore, the concentration of ECL probes is not independently linearly related to ECL intensity. Some specific properties need to be met to obtain an accurate and feasible potential-resolved multimarker ECL analysis system without signal interference: (1) Each luminophore should have a separate ECL response that is potentially and originally independent. (2) RET and chemical reaction cross-talk should not exist between luminescent systems. (3) Co-reactants must not interfere with each other.

### Luminophore-Co-reactant Pairs in Simultaneous Multimarker Detection

#### Bilateral Anodic-and-Cathodic Potential Luminescence

**Figure 6:**
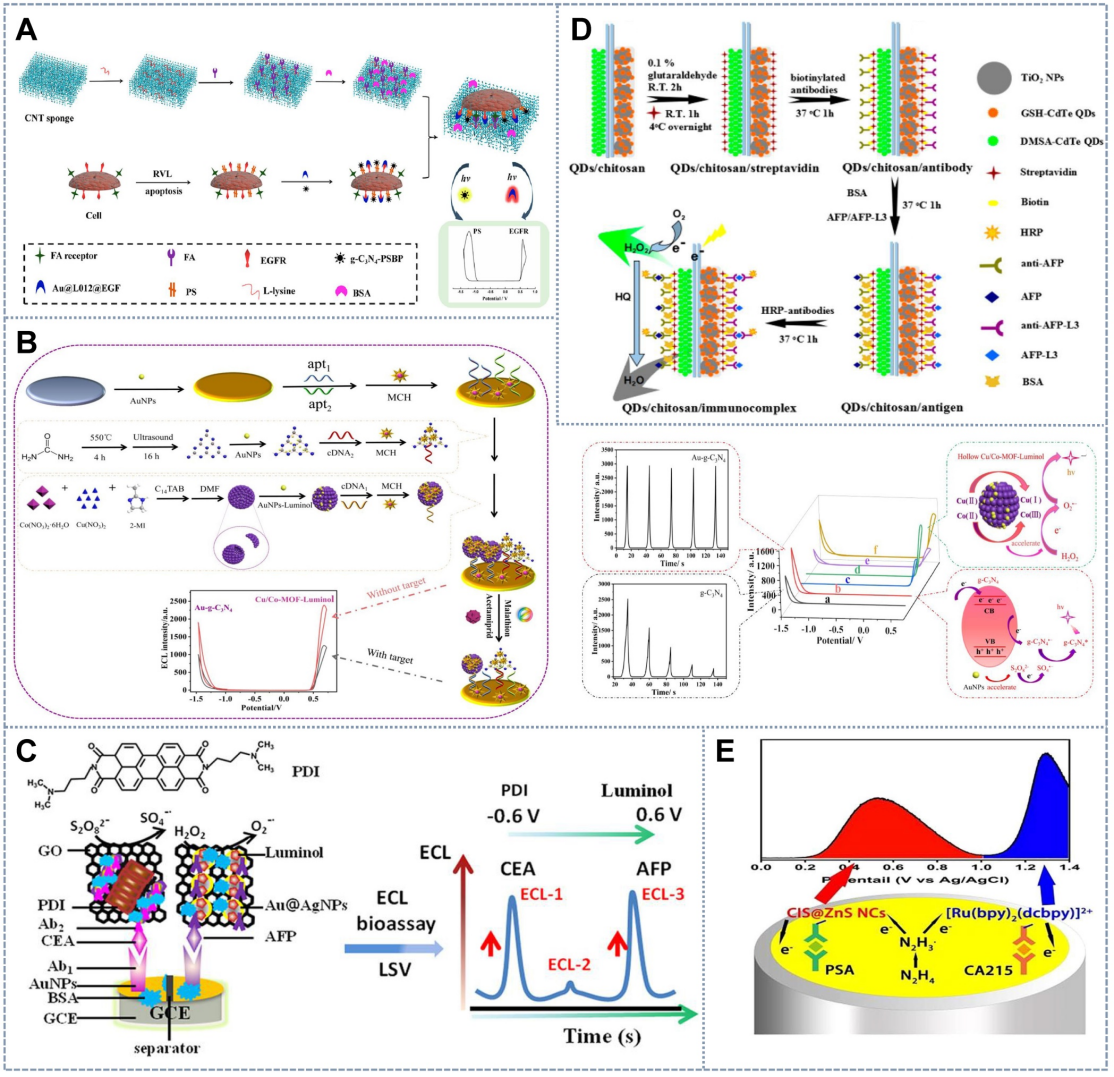
Schematic of sensors for simultaneous multimarker detection based on bilateral anodic-and-cathodic-potential luminescence with **(A)** L012 and g-C_3_N_4_ as the luminophore pairs for the *in situ* detection of apoptosis factors. Adapted with permission from 26, copyright 2019 American Chemical Society. **(B)** Luminol and g-C_3_N_4_ as the signal probe. Adapted with permission from 101, copyright 2021 Elsevier B.V. Biosensors based on unilateral low-potential luminescence with **(C)** the low-potential cathode luminophore PDI and co-reactant K_2_S_2_O_8_. Adapted with permission from 114, copyright 2019 American Chemical Society; **(D)** homogenous luminophores GSH-CdTe QDs and DMSA-CdTe QDs sharing the co-reactant H_2_O_2_. Adapted with permission from 115, copyright 2021 Elsevier B.V.; and **(E)** CIS@ZnS NCs emitting at the ultralow potential of 0.30 V and (Ru(bpy)_2_(dcbpy))^2+^ as luminophores. Adapted with permission from 16, copyright 2021 Elsevier B.V.

Luminophores of vastly different excitation potentials should be chosen for multiplex ECL assays to avoid the overlapping and indistinguishability of two ECL peaks and to enable the complete resolution of the concentration information of multiple markers. Therefore, an anode luminophore, such as luminol and Ru-related complexes 88, and a cathode luminophore, such as QDs 18,45, perylene tetracarboxylic acid, and g-C_3_N_4_ 5,100, are usually applied in previously reported potential-resolved multilabel ECL biosensors. Zhao *et al.* 99 constructed a novel potential-resolved ECL strategy based on the luminophore pair ABEI/O_2_ (+0.7 V) and g-C_3_N_4_/S_2_O_8_^2-^ (-1.5 V). Their emission potentials can be clearly distinguished, and no RET occurred between them. Given the absence of cross-interference and competition between ABEI's co-reactant dissolved oxygen and g-C_3_N_4_'s co-reactant K_2_S_2_O_8_, ABEI and g-C_3_N_4_ have become an ideal combination for multimarker detection. Similarly, Liu *et al.* 26 introduced the luminol analogue 8-amino-5-chloro-7-phenylpyrido(3,4-d)pyridazine-1,4(2H,3H)-dione (L012)(+0.6 V) and g-C_3_N_4_ (-1.2 V) as a luminophore pair to construct a new *in situ* detection system for the degree of cell apoptosis, which is induced by the reduction in the expression of epidermal growth factor receptor (EGFR) and the increased eversion of phosphatidylserine (PS) on the cell membrane (Figure 6A). Epidermal growth factor and the peptide PSBP were functionalized with Au@L012 and g-C_3_N_4_ as the ECL probes for the recognition of EGFR and PS expression on the cell surface. Thus, the degree of apoptosis was clearly reflected by the change in the two well-separated ECL signals from g-C_3_N_4_ and Au@L012. This strategy therefore provides an accurate and reliable way to investigate apoptosis. Similarly, by employing analogical emitters, Liu *et al.* 101 introduced a hollow Cu/Co MOF with effective catalytic performance and loading capacity to construct an ECL aptasensor for the simultaneous detection of acetamiprid and malathion. In this system, luminol and g-C_3_N_4_ NSs were used as the signal probe (Figure 6B). The hollow Cu/Co MOF was applied to load the luminol and significantly improved the ECL signal by catalyzing H_2_O_2_ to produce additional O^2•-^. Meanwhile, the material's conductivity was effectively guaranteed because electron mass transfer resistance was avoided by the low density of Cu/Co MOFs. The constructed ECL aptasensor not only eliminated cross-talk between luminescent systems but also exhibited the excellent specificity and sensitivity of 0.015 and 0.018 pM(S/N = 3), respectively, and a low detection limit. The integration of anodic and cathodic ECL luminophores effectively expanded the potential window, which is crucial for improving resolution for ECL multiple target determination. Moreover, highly distinguishable anodic-and-cathodic on-set potential luminophore pairs have been developed ingeniously and flexibly to achieve the simultaneous detection of single cells and cell surface markers. He *et al.* 102 proposed a novel strategy that used capture DNA-hybridized aptamers intercalated with Ru(phen)_3_^2+^ ECL probes to detect cancer cells at positive potential and that applied concanavalin A-conjugated AuNP-modified graphite-C_3_N_4_ to evaluate N-glycan at the cell surface at negative potential. The ratio of the ECL intensity between the negative potential and positive potential (ΔECLn/ΔECLp) enabled simultaneous cytosensing and cell surface N-glycan evaluation. This situation significantly facilitated the understanding of the biological processes related to complex native glycan and promoted the elucidation of N-glycan-related diseases and the clinical diagnosis of physiological processes.

#### Unilateral Low-potential Luminescence

As described above, given the limited potential scanning window and the easy overlapping of luminous peaks, luminophore pairs with independent ECL at the anode and the cathode are generally adopted to improve resolution strategies. However, the majority of anodic and cathodic ECL luminophores need different co-reactants, and their inevitable side reactions over a wide potential scan interfere with the ECL process 103. Potential-resolved luminophore pairs sharing the same co-reactant and emitting over a narrow potential scan (i.e., the single anodic or cathodic scan] are preferred given the above issues. Earlier studies focused only on a few types of these multiplex systems given their following limitations: (1) The potential window tolerable for bioassays is relatively narrow, whereas the complete potentially distinguishable multiple ECL signals under single anodic or cathodic scanning usually extends to fairly high voltages because of the wide triggering-potential peak of some ECL luminophores 16. (2) Although many ECL luminophores, such as cyclometalated Ir (III)/Ru(II) complexes, exhibit flexible and adjustable luminescence potentials and wavelengths as a result of introducing substituents on their cyclometalated ligands or changing their coordination ligand, they are mainly applied in organic solutions and cannot be used as labels for multitarget bioassays. (3) The redox potential of traditional ECL luminophores/co-reactants is usually high (greater than -1.2 V or even -2 V vs Ag/AgCl). However, at such high cathode potentials, the dissolved oxygen in the electrolyte produces ECL, which causes background signals and reduces sensitivity 104-110.

Although developing ECL with good potential resolution in a limited potential window is very difficult 111,112, several methodologies for multicomponent analysis have been exploited and shown extremely high sensitivity and accuracy. Gao *et al.* 113 synthesized the cyclometalated Ir (III) complex (dfppy)_2_Ir (dcbpy)PF_6_ with high quantum yields and good solubility in aqueous solution. They combined this complex with Ru(bpy)_2_(mcbpy-O-Su-ester)(PF_6_)_2_ in the presence of tripropylamine to acquire well-separated strong ECL emissions at +0.9 and +1.4 V vs Ag/AgCl and the large difference of the ECL peak potential (~ 0.5 V) at the gold electrode. Song *et al.* 114 synthesized a new type of low-potential cathode luminophore, namely, *N*,*N*-bis-(3-dimethylaminopropyl)-3,4,9,10-per tetracarboxylic diimide (PDI), with K_2_S_2_O_8_ as the co-reactant. This luminophore can produce ECL responses at -0.6 V (Figure 6C). In addition, in contrast to other single-emission emitters, the J-type PDI dimer formed by the excitation of PDI in the ECL reaction had double ECL emission with a wavelength peak of 717-718 nm at -0.25/-0.26 V, thus showing a unique built-in self-calibration capability for accurate quantitative and biological imaging analyses. In this study, PDI and luminol were used as the potential-resolved luminants to measure carcinoembryonic antigen and AFP simultaneously over the low potential range of -0.6 V to 0.6 V. In combination with the monomercapto-stabilized CdTe QDs (GSH stabilized-CdTe (GSH-CdTe QDs)) that are commonly used as ECL luminophores (-1.25 V), Liu *et al.* 115 introduced 2,3-dimercapto-succinic acid-stabilized CdTe (DMSA-CdTe) QDs into a multiplex ECL system as another signal probe with a relatively low emission potential (-0.89 V vs Ag/AgCl) (Figure 6D). Given the homogeneity of the two luminophores, DMSA- and GSH-CdTe QDs had similar luminescent mechanisms in an aqueous environment. The emissions of the two luminophores were assisted by H_2_O_2_, the collective co-reactant that was self-produced *in situ*, thus forming a benign and simple nano-ECL multimarker detection system. Li *et al.* 16 realized potential-resolved ECL in the single anodic region by using CIS@ZnS NCs and (Ru(bpy)_2_(dcbpy))^2+^ as luminophores and N_2_H_4_ as the co-reactant in aqueous solution. In this system, CIS@ZnS NCs/N_2_H_4_ emits at the ultralow potential of 0.30 V, whereas Ru(bpy)_2_(dcbpy)^2+^/N_2_H_4_ presented emission from 1.00 V to 1.40 V (Figure 6E). The large separation in onset potential led to a high resolution of 2.60 V that far exceeded the 1.50 V threshold for the total baseline separation. The good resolution of this strategy in the monodispersed and immune-sensing states (baseline separation, Rs ≥ 1.5) proved its usefulness in dual-component assays for practical applications.

### Anti-cross-talk Strategy in the Simultaneous Detection of Multiple Markers

In ECL multimarker analysis, the potential-resolved ECL emitter pairs that can avoid mutual interference between the co-reactants and RET between luminophores are very limited. A series of strategies have been reported to solve the cross-talk between luminescence systems. Moreover, some ECL platforms for the detection of multiple markers have been proposed to expand the application potential of multimarker detection.

#### Spatial-resolved System

**Figure 7:**
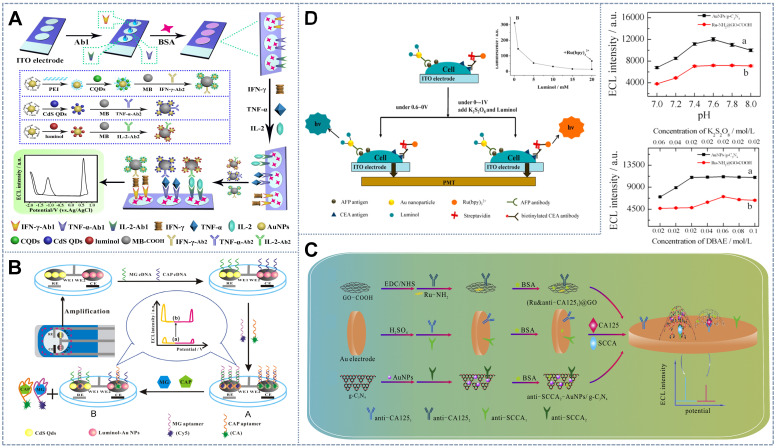
Schematic of anti-cross-talk multidetection sensors based on spatial-resolved strategy with **(A)** an ITO electrode with three spatially resolved regions for the detection of three biomarkers. Adapted with permission from 116, copyright 2017 American Chemical Society; **(B)** excessive DBAE. Adapted with permission from 117, copyright 2017 Elsevier B.V.; **(C)** a novel double working electrode aptamer sensor array on a SPCE. Adapted with permission from 118, copyright 2015 Elsevier B.V.; and the concentration-controlled strategy with **(D)** the high concentration of luminol. Adapted with permission from 119, copyright 2014 American Chemical Society.

The resonance transfer of energy between light-emitting systems can be reduced by separating these systems from each other by a certain distance in space. The distance between different ECL probes must exceed nanometers to eliminate interference between ECL probes on one electrode. Zhou *et al.* 116 introduced a patterned indium tin oxide (ITO) electrode with three spatially resolved regions (with the spacing of 1.5 mm) and combined the three latent tuberculosis infection (LTBI) marker antibodies IFN-γ-Ab1, TNF-α-Ab1, and IL-2-Ab1 on the three spatially resolved regions. By using luminol, carbon QDs, and CdS QDs as potential-resolved ECL probes and K_2_S_2_O_8_ (0.1 M) and H_2_O_2_ (10 mM) as co-reactants, ECL emission peaks with the peak voltages of +0.6, -1.2, and -1.8 V (vs Ag/AgCl) were obtained, thus achieving the simultaneous detection of the three types of LTBI markers (Figure 7A). In accordance with the same principle, Feng *et al.* 117 established a novel double-working electrode ECL aptamer sensor array on a self-made SPCE (Figure 7B). The SPCE substrate was composed of two carbon working electrodes (WE1 and WE2). The aptamer sensor array not only had multiple detection functions, it also avoided the cross-talk and cross-reaction between light-emitting systems. On the electrode surface, the complementary DNA sequence (MG cDNA or CPA cDNA) fixed on the luminophore (CdS QDs (-1.15) or luminol-Au(+0.6V)) hybrids with the aptamer modified by the quencher Cy5 (quencher for CdS QD) or chlorogenic acid (CA, quencher of L-AuNP) formed two double-stranded structures on WE1 and WE2. In the presence of the target malachite green (MG) and CAP, the combination of MG and CAP with the aptamer resulted in the cleavage of the corresponding double-stranded structure, making Cy5 and CA separate from SPCE such that the ECL intensities of the cathode and anode increased simultaneously. This strategy could be used to detect MG and CAP with linear ranges of 0.1-100 and 0.2-150 nM, respectively, and detection limits of 0.03 and 0.07 nM, respectively.

#### Concentration-controlled Measures

In addition to eliminating cross-talk between light-emitting systems by means of spatial resolution, the influence of cross-reactions can be avoided by controlling the amount of luminophores or co-reactants that can cause cross-talk. Guo *et al.* 118 proposed the combination of Ru-NH_2_ and AuNPs/g-C_3_N_4_ as two potential-resolving luminous bodies and DBAE and K_2_S_2_O_8_ as the co-reactants with two strong and stable ECL emissions at 1.25 and -1.3 V, respectively (Figure 7C). However, during the positive potential scanning, DBAE was oxidized and deprotonated on the electrode into the strongly reduced intermediate DBAE·, which not only reduced the electrode oxidation product Ru^3+^ to produce ECL emission but also reacted with the reduction product of K_2_S_2_O_8_, the co-reactant of g-C_3_N_4_. Therefore, the concentration of K_2_S_2_O_8_ interfered with the production of the ECL signal by the luminophore Ru-NH_2_ and the co-reactant DBAE. To reduce the system error, Guo *et al.* added excess DBAE such that the consumption of DBAE by K_2_S_2_O_8_ was negligible, thereby eliminating the cross-reactions between the co-reactants of the dual luminescence system. Additionally, Han *et al.* 119 used Ru(bpy)_3_^2+^ and luminol as the cathode and anode ECL probes on a single electrode to measure two antigens on the cell surface (Figure 7D). Although the potential-resolved ECL based on the luminol/Ru(bpy)_3_^2+^ system is feasible in principle, luminol cross-reacts with S_2_O_8_^2-^, which is the co-reactant of Ru(bpy)_3_^2+^, and produces luminescence at the same potential as Ru(bpy)_3_^2+^. The overlapping of the two luminescence peaks resulted in the nonlinear relationship between the logarithmic value of the concentration of the luminescent probe and the intensity of the lighting signal. To overcome the above problem, Guo *et al.* introduced high concentrations of the water-soluble luminol, which caused the self-quenching of its reaction with S_2_O_8_^2-^ under negative potential. This approach thereby greatly reduced the signal cross-talk generated by the cross-reaction of luminol and Ru(bpy)_3_^2+^ with their mutual co-reactants at each other's luminous potential.

### Real-time and On-spot Detection

**Figure 8:**
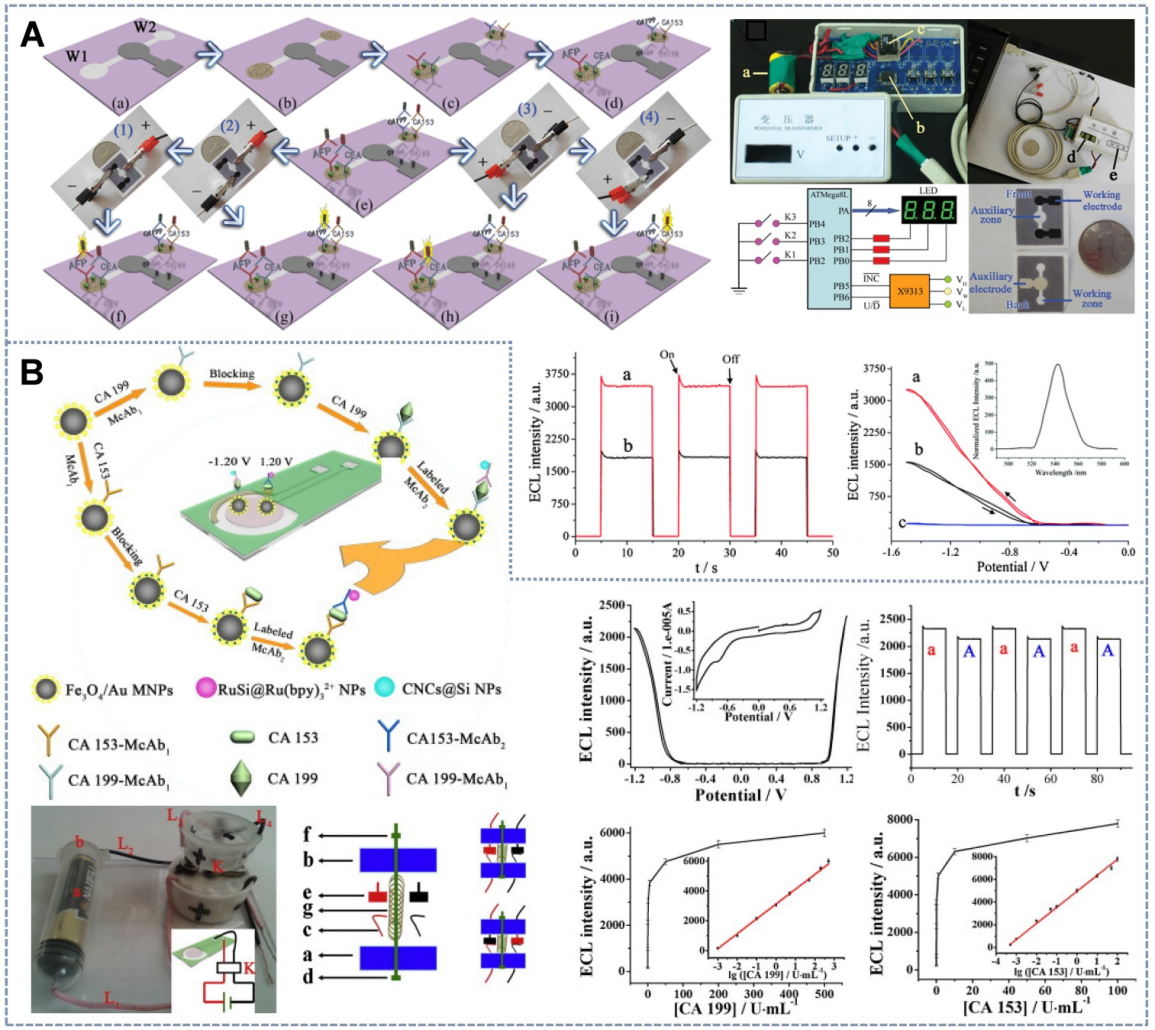
Schematic of biosensors with real-time and on-spot detection based on **(A)** a battery-based microfluidic paper. Adapted with permission from 122, copyright 2012 Royal Chemistry Society. **(B)** Automatically toggled switch. Adapted with permission from 123, copyright 2013 Elsevier B.V.

On-spot detection is a popular research direction for biosensors. The development of real-time detection and easy-to-carry biosensors is a prerequisite for the development of the Internet-of-Things 120,121. Microfluidic paper-based analytical devices (μ-PADs) and paper-based analytical devices are considered excellent equipment for point-of-care testing because of their low cost, ease of use, ability to save reagents, portability, and disposability. These paper-based devices can freely adjust the fluid flow driven by the inherent capillary force after sample loading and immune response, thus allowing simultaneous parallel and multiple measurements. Wang *et al.* 122 introduced the potential-resolved multimarker detection mode into μ-PAD devices for the first time (Figure 8A). Ru(bpy]_3_^2+^(+1.2 V) and carbon nanodots (CNDs) (-1.2 V) fixed on a screen-printed carbon working electrode were used as ECL probes. The two double-probe-labeled working electrodes enabled the simultaneous detection of four tumor markers. In this system, the traditional electrochemical workstation was replaced with a battery, and the output voltage of the battery was precisely controlled by a low-cost and simple voltage controller. In accordance with the detection mode of potential discrimination, multiple immunoassays can be performed in one work area by only adjusting the direction of the positive and negative connections between the paper detection platform and the power supply device. This battery-based microfluidic ECL immunosensor provides a new strategy for high-throughput, low-cost, sensitive, and automated multiple immunoassays and instant diagnosis. On the basis of this research, Li *et al.* 123 designed a self-assembled toggle switch that can automatically switch the positive and negative potentials of the working electrode (Figure 8B). By using Ru(bpy)_3_^2+^-conjugated silica NPs as the anode ECL label (+1.20 V), carbon nanocrystals deposited silica nanoparticles (CNCs@Si NPs) as the cathode ECL label (-1.20 V), and replacing the traditional three-electrode, ceramic or paper-based devices by transparent, low-cost ITO based two-electrode system, a low-cost, portable and battery rechargeable constant voltage ECL multiple immunosensor was developed with an output voltage of 1.20 V, which was employed to detect CA 153 and CA 199 with detection limits of 1.3×10^-4^ U ml^-1^ and 2.3×10^-4^ U ml^-1^, respectively The constant potential ECL system based on rechargeable batteries showed good stability and repeatability. Furthermore, cheap and portable multimarker real-time detection systems can be obtained by replacing the expensive electrochemical workstations in some ECL systems with rechargeable batteries.

## Potential-resolved Multicolor ECL

As discussed above, the potential-resolved strategy could greatly simplify operation and reduce the analytical time of ECL detecting systems because it does not require a filter or beam splitter, unlike other resolved strategies, such as the spatial-resolved strategy or the spectrum-resolved strategy. Nevertheless, with the expansion of the requirements for the number of output signals, the application of the potential resolved strategy alone can no longer meet demands because of the following restrictions: (1) When the electrode potential exceeds the corresponding threshold value, the ECL of each luminophore is persistently generated 22. (2) The pairing of ECL probe pairs with indistinct onset-potential differences results in poorly resolved emission 134. One general solution to this current challenge is to combine a potential-resolved strategy with spectrum-resolved strategy 135,136. The spectrum-resolved technique, in contrast to the potential-resolved strategy, distinguishes the signal based on the varied emission wavelengths between emitters 14. Therefore, when the peak potential of the two emitters is relatively close to causing the signal peak to overlap, the luminous intensity at distinct wavelengths may be utilized to identify the analyte 22. Furthermore, the luminous wavelength of the luminophor is mostly unaffected by the potential or other substances in the system 137. Consequently, the combination of potential resolution with spectral resolution can broaden the potential sweep and enable the simultaneous detection of multiple markers. Following the development of monochromatic electrochemiluminophores of different wavebands 138 and a CCD-based ECL spectrum analyzer 139, the key obstacles in spectrum-based ECL could be resolved, which makes it qualified for the potential-resolved and color-selective ECL models. This strategy is also known as “potential-resolved multicolor electrochemiluminescence (PRMCECL)”, specifically, multicolor ECL emissions under different potentials during an electrochemical scan 140. The application of the PRMCECL strategy in ratiometric 141,142 and multiplex ECL assays 134 has attracted substantial attention given that it either provides additional signal information for self-calibration or enables the concurrent detection of multiple analytes. In addition, on the basis of its sufficiently powerful optical signal output, it can be utilized to construct and optimize visualized ECL sensors, which have now become popular for their direct and intuitive data readout pattern that is visible to the naked eye. Given the human eye's low sensitivity to the resolution of monochromatic intensity, which leads to low detection sensitivity and the formation of erroneous conclusions, many visual signals are now only employed for quantitative or semiquantitative analysis 143. The luminescence color of luminophor pairs possessing potential-resolved and wavelength-resolved properties can be used to determine analyte concentration quantitatively, which is critical to the development of multichannel bioassays and to reduce detection costs 14,144,145. In accordance with different luminescence and reaction pathways, PRMCECL can be divided into concomitant metal complexes and single luminescent clusters, of which concomitant metal complexes have two luminous pathways: co-reactant multicolor ECL and annihilation multicolor ECL 116,145-148.

### Concomitant Metal Complexes

Metal coordination complexes have attracted tremendous research attention and have been successfully developed in the PRMCECL system due to their remarkable features, such as rapid response time; wide dynamic range; low sample consumption; high on-board stability; excellent precision; and, most importantly, zero-background sensitivity 135, 149-153. When the ECL signal output must be clearly spectrally resolved to perform multiple assays, cyclometalated Ir complexes with emission maxima spanning the entire visible spectrum are used 154-159, thus opening up new possibilities for color-tunable light-emitting devices and simultaneous multianalyte detection based on multiple spectrally distinct ECL species 135,160-163.

**Figure 9:**
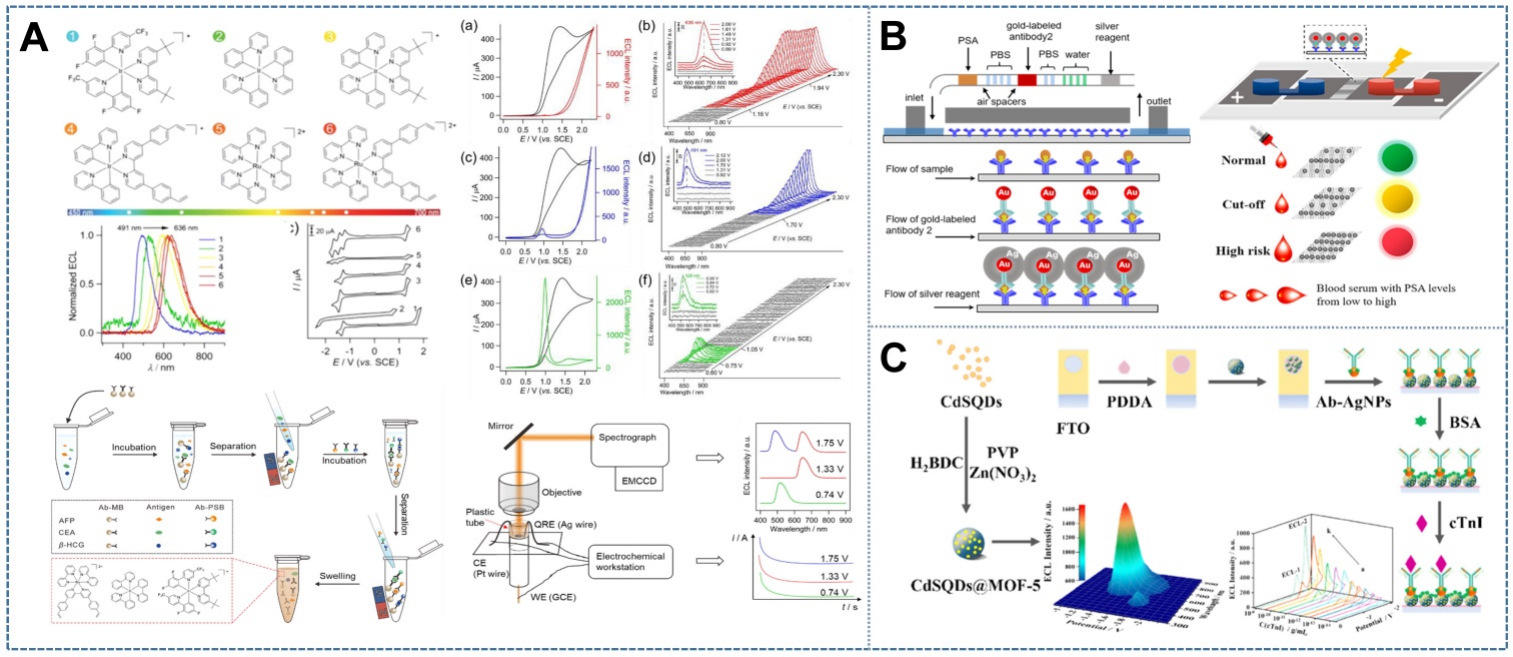
Schematic of the application of the PRMCECL strategy in **(A)** MIA by using several self-synthesized concomitant metal complexes with expanded spectral coverage. Adapted with permission from 134, copyright 2018 American Chemical Society. **(B)** Immunosensor visualization by regulating the interfacial potential (Δϕa) at the poles of BPE. Adapted with permission from 183, copyright 2017 American Chemical Society. **(C)** Ratiometric biosensor based on the PRMCECL nanoluminophore CdSQDs@MOF-5. Adapted with permission from 141, copyright 2020 American Chemical Society.

The PRMCECL strategy can be divided into three categories in accordance with the choice and synthesis of the concomitant metal complex and the construction of the ECL system. The first type of multicolor ECL is a mixture consisting of Ru(II) and Ir(III) complexes. For example, given that the luminescence of Ru(bpy)_3_^2+^ and its derivative complexes is characterized by a broad spectral line width (FWHM of 60-80 nm) 134,164, long radiative decay lifetime (typically microseconds) 165, low photoluminescence (PL) quantum yield (PLQY, ~ 4.2% for Ru(bpy)_3_^2+^), and difficult multicolor detection, Guo *et al.* 134 expanded the spectral coverage of the concomitant metal complexes by synthesizing several Ru and Ir complexes with distinguishable ECL emission wavelengths that ranged from 491 nm to 636 nm (Figure 9A). The ECL performance of the mixtures was investigated by using multiple ECL readout modes (intensity, spectrum, and imaging measurements). A spectral peak separation of up to 145 nm was found between the cyan-ECL-generating species Ir(dFCF_3_ppy)_2_(dtbbpy)^+^ and the red-ECL-generating species Ru(bpy)_2_ (dvbpy)^2+^. Therefore, the mixed electrochemiluminescent system achieved spectrally resolved ECL generation. Potential-resolved ECL emissions were obtained by using Ir(ppy)_3_, a green-ECL luminophore with self-annihilating ECL behavior at high potentials, for the quenching of (Ir(ppy)_3_)^*^ by TPrA^+•^ with either Ru(bpy)_2_(dvbpy)^2+^ or Ir(dFCF_3_ppy)_2_(dtbbpy)^+^. A multiplex immunoassay (MIA) free of spatial spotting antibodies on plates or substrates was ultimately devised by combining luminophore-loaded polymer beads with the homogeneous sandwich immunoreaction method. The simultaneous recognition of three antigens through the use of the potential and spectrum dual-resolved ECL as the readout signal illustrated the vast potential of this approach for multiplex ECL detection. The second type of multicolor ECL is from multimetal (e.g., Ru^2+^ and Ir^3+^) centers within a single molecule. Previously reported work usually used Ir and Ru complexes as ECL emitters and regulated the applied potential to achieve spectral resolution 135. However, this strategy relies on intermolecular reactions between two emitters and can be easily interfered with by other substances in the system because of steric hindrance and energy loss 167. This problem can be solved by combining two luminophores into one molecule and relying on intramolecular interactions. For example, Peng *et al.* creatively designed and synthesized heterodinuclear (bpy)_2_Ru (bpy)(CH_2_]_n_(bpy)Ir(df-ppy)_2_]^3+^ (n = 10, 12, 14) complexes that combined Ir and Ru in a single molecule. The color of the light can be changed by regulating the applied potential in accordance with the intramolecular interactions between the Ir and Ru moieties. When the scan potential ranged from 0.55 V to 0.95 V, the ECL intensity at 543 nm from the Ir moiety and that at 618 nm from the Ru moiety showed good linear calibration curves. When the scanning voltage ranged from 1.0 V to 1.6 V, the emission from the Ru moiety increased rapidly, whereas the Ir moiety had little or no emission due to the RET between the Ir and Ru moieties. This strategy can also help find new ECL luminophores. Sun *et al.* combined luminol with the classical dyes 7-(diethylamino) coumarin-3-carboxylic acid (CO) and 3- (4-amino-1,8-naph-thalimido) propanoic acid (NA). The dyes were ECL inactive originally, but because of the energy resonance transfer between luminol and the dyes, the LU-CO and LU-NA complexes exhibited emission under the potential of 0.5 V at 481 and 540 nm, respectively 168. The third type of multicolor ECL originated from light-emitting devices, which combine solid-state ECL materials (metal complexes) with organic light-emitting diodes (e.g., small organic molecules and polymers). Sandwich-structured organic light-emitting diodes (OLEDs) and solid-state electrochemiluminescence devices (SECLDs) that usually use Ru tribipyridine as the luminescent molecule have received considerable attention. However, their operation mechanisms have many differences, such as carrier injection and hole and electron mobility. De Cola *et al.* designed a new light-emitting device that combined OLEDs and SECLDs together to obtain some insight into equipment characteristics, such as electronic injection and recombination, and seek new ways for instrument optimization. In their work, 3% wt (Ru(bpy)_2_ (dimbp))(PF_6_)_2_ in pyridine solution, aluminum tris-(8-hydroxyquinoline)(Alq_3_), and *N*,*N*ʹ-diphenyl-benzidine were loaded on an ITO in sequence. The ECL emission centered at 625 nm was acquired under reverse bias (-10 V). The color of the light changed from yellow to green to white over time under forward bias (+9V) 169.

ECL emission principles are often categorized into two general pathways: annihilation and co-reaction. Both pathways play an important role in PRMCECL. The first co-reactant multicolor ECL was reported by Richter *et al.* 135, who utilized the combinations of (Ru (bpy)_3_)^2+^ (I_max_ = 620 nm) and either fac-Ir(ppy)_3_ (I_max_ = 517 nm) or (Ir (df-ppy)_2_(pic)) (I_max_ = 498 nm) with TPA as the oxidative-reductive co-reactant that generated simultaneous ECL from the two luminophores (albeit with considerable overlap between emission bands) at the single applied potential of 0.8 V. Doeven *et al.* 170 demonstrated that by scanning or stepping from low to high electrode potentials, the mixtures of electrochemiluminophores may also be identified selectively on the basis of their unique redox energies. For example, in the presence of a green-emitting (Ir(df-ppy)_2_(BPS))^-^ complex, a red-emitting (Ru(bpy)_2_(L))^2+^ complex was stimulated preferentially at a low electrode potential (1.05 V vs. Fc^0/+^), and simultaneous ECL from the two complexes was detected at a high electrode potential (1.30 V vs. Fc^0/+^), thus allowing the total emission color to be adjusted from red to yellow-green. Similarly, Schmittel *et al.* 171 demonstrated that the ratio of emissive transitions within a non-Kekul e-structured trinuclear Ir(III)-Ru(II)-Ir(III) species was dependent on the applied potential. The wavelength of maximum ECL intensity could be tuned between 649 and 611 nm as the anodic scan range was increased. However, these findings cannot realize complete and reversible switching between the emissions from two distinct ECL luminophores due to the difficulty in switching off the low-potential electrochemiluminophore at potentials where the high-potential luminophore is excited. Such a situation may induce the unwanted interference of the emissions of two luminophores in multiplex ECL-based assays. Therefore, by exploiting the switch-off mechanism of Ir(ppy)_3_ at high overpotentials likely through the oxidative quenching of the excited (Ir(ppy)_3_)^*^ state, Doeven *et al.* 170 selectively elicited green ECL from Ir(ppy)_3_ at low potentials and either red or blue ECL from (Ru(bpy)_2_(L))^2+^ or Ir(df-ppy)_3_ at high potentials. Although two-component mixed ECL systems have been extensively explored, their application in high-throughput testing remains limited. As a result, a system with additional luminators and resolved colors should be developed. However, the development of a three-color ECL system with the concomitant metal complex is hampered by the broad spectral distributions and significant spectral overlaps (i.e., 26% of the integrated peak area of Ru(bpy)_3_^2+^ (max = 620) and fac-Ir(ppy)_3_ (max = 520 nm)) of the conventional Ru- and/or Ir-complexes. To solve this problem, Doeven *et al.* 172 modified the ligand structure to control the border orbital energies, which influence the complexes' spectroscopic and electrochemical characteristics. Specifically, they created a deep red emitter [(Ru(bpy)_2_(dm-bpy-dc)]^2+^, max = 685 nm] by adding two electron-withdrawing methyl ester groups on one of the (Ru(bpy)_3_)^2+^ bpy ligands, thus producing a bathochromic shift in emission while preserving a favorable oxidation potential for ECL 173. Similarly, the addition of fluorine groups to the phenyl rings of Ir(ppy)_3_ caused a large hypsochromic shift by stabilizing the HOMO level of the mixed metal-ligand, resulting in a suitable blue emitter (Ir(df-ppy)_3_, λmax = 495 nm) 170. A compound containing a triazolylpyridinato ligand (Ir(df-ppy)_2_[ptp], λmax = 463 and 492 nm), which further blue-shifted the emission and gave a three-fold boost in ECL intensity, was also created 174. With these changes, spectrally resolved red and blue emitters (overlapping in the integrated peak area of just 5%) were obtained. In addition, a third concomitant electrochemiluminophore green-emitter (Ir(ppy)_3_) was used to extend the system for three-channel detection by taking advantage of the different potentials required for electrochemical excitation 122,160,161 and the recently discovered selective ECL quenching of the co-reactant Ir(ppy)_3_ at high overpotentials 170. Then, by employing separate applied potentials, the first multicolor ECL system with three effectively resolved emitters was demonstrated in combination with the intrinsic color selectivity of a standard digital camera.

Another important concept is the annihilation route, in which oxidized and reduced species are produced at two separate electrode potentials and then comproportionated to produce an emissive excited state to provide an alternative to multicolor ECL. The mixed annihilation ECL of metal chelates containing more than one organic compound or a transition-metal chelate with a nonemissive organic compound 175 to generate the relative intensity of multiple luminophores (and thus overall emission color) that can be controlled initially by selecting the electrochemical and spectroscopic properties of the complexes, as well as the applied electrochemical potentials, was explored. The factors controlling the ECL emission color in mixed annihilation ECL are (1) the relative concentrations of two complexes 176-178; (2) the relative ECL quantum yields; and (3) the efficiency of various energy transfer pathways 179, such as RET or electron transfer/exchange, among the numerous oxidized, reduced, ground, and/or excited states of the complexes that are probably determined by the numerous closely spaced reductions and oxidations of the mixed system. Kerr *et al.* 175 created the mixed annihilation ECL system of transition-metal complexes (combining (Ru(bpy)_3_)^2+^ with a range of Ir(III) complexes), wherein the relative strength of the emissions could be adjusted by the applied voltage to alter the overall hue of the luminescence. This effect is due to the generation of distinct redox forms by the complexes that altered the energetics of the light-producing processes. In a subsequent study, Swanick *et al.* 180 investigated the ECL of a soft salt Ru(II)-Ir(II) complex 181, which contained a (Ru(dtb-bpy)_3_)^2+^ cation (dtb-bpy = 4,40-di-t-butyl-2,20-bipyridine) and two (Ir(ppy)_2_ (CN)_2_)^-^ anions (where ppy = 2-phenylpyridine), in solution. They reported that in contrast to the PL of the soft salt under nearly comparable conditions, ECL originated entirely from the Ru(II) complex (i.e., no emission from the Ir(III) complex). Swanick *et al.* 179 attributed their findings to the soft salt's ion pairing interactions, which assisted efficient quenching inside the mixed annihilation ECL system. Subsequently, Kerr *et al.* 182 reconciled these contradictory findings by investigating the concentration effects and energy transfer in mixed annihilation ECL. They also introduced a novel three-dimensional representation of the phenomenon (annihilation ECL intensity versus emission wavelength and the applied reduced potential), as well as a simple graphical depiction of the energetics of annihilation and co-reactant ECL systems, for the investigation of electron-transfer quenching pathways. They confirmed that the contradictory findings of previous studies can be largely attributed to differences in the relative concentrations of electrochemiluminophores; the relative ECL intensities of individual and mixed annhiliation ECL reactions; and the efficiency of various energy transfer pathways, which are likely the result of a combination of several concomitant pathways that may include RET or electrification.

For the construction of PRMCECL biosensors, the bipolar electrode, a conductive material that promotes electrochemical reactions at its extremities under sufficient driving potential 183, has been widely utilized for multicolor visualization 184. Selective ECL excitation is carried out by regulating the interfacial potential (Δϕa) at the poles of BPE on the premise of the potential resolution of the emission color of concomitant luminophores 185. Wang *et al.* 183 devised the first PRMCECL device for biological analysis on the basis of closed BPE by utilizing a mixture of the red and green luminophores Ru(bpy)_3_^2+^ and Ir(ppy)_3_ with TPrA as the co-reactant (Figure 9B). A silver bridge with PSA-concentration-dependent properties was built in the gap between the BPEs to modulate resistance. By moderating the interfacial potential difference (Δϕa), the kinetics of Faradic reactions at the surface of the BPE would be altered, finally leading to a visible three-color change (green-yellow-red) at the anode. These eye-catching color changes could effectively reflect the cut-off values (4.0 and 10.0 ng/mL) of human PSA, thus demonstrating that the clinical use of the proposed PRMCECL biosensor is feasible and reliable. On this basis, Luo *et al.* achieved the rapid visual detection of the foodborne pathogen *Salmonella typhimurium* in a complex food matrix by taking advantage of the separate reservoir of the closed BPE system 186, in which the analytes were not in direct contact with the photoactive molecules in the complex reaction systems of the anode, thus efficiently avoiding mutual interference. The presence of *S. typhimurium* on the BPE cathode increased the resistance of the cathode. This effect induced the emission color of the Ir(ppy)_3_ and Ru(bpy)_3_^2+^ complexes to switch from dark-orange to yellow to forest-green. This phenomenon enabled the determination of *S. typhimurium* by the naked eye at the fairly low limit of detection of 10 CFU/mL in a 25 mL or 25 g sample within 0.5 h. Furthermore, in resistance-resolved BPE-ECL, cathode reactions, which alter the Faradaic current through BPE, could also be observed on the basis of the emission color of the anode. Wang *et al.* 145 introduced a special excitation potential combination containing Ru(bpy)_3_^2+^ with the luminous peak voltage in between those of Ir(df-ppy)_2_(pic), which enables the bidirectional change of the emission color (blue-green to red to blue-green) while either decreasing or increasing the Faradaic current. This array could simultaneously detect PSA, circulating microRNA-141, and the small molecular marker sarcosine with a single DC power supply. Therefore, the BPE-ECL device may greatly expand the construction strategies of PRMCECL biosensors, which have considerable potential applications in clinical diagnostics for the fast and intuitive judgment of multiple analytes.

### Single Luminescent Clusters

In the last several years, PRMCECL nanoluminophores and QDs have emerged and shown promising applications in sensitive and accurate bioassays, bioimaging, and multicolor-emitting devices.

Three types of potential-resolved multicolor ECL nanoluminophores have been reported. The first type is the ECL nanoluminophores without functionalization or hybridization. On a negative potential scan, Zhang *et al.* presented a dual-peak ECL system of carbon dots in ethanol with tetrabutyl ammonium bromide as the co-reactant; this system was used to identify iron ions via the internal standard method 187. The second type is ECL nanoluminophores based on the hybridization of two nanomaterials. Ding *et al.* synthesized PbS nanocrystals capped with boron-dipyrromethene dye to produce highly efficient dual ECL emissions with tripropylamine as the co-reactant in organic solvents 188. However, the multicolor ECL emissions generated in organic solvents were unsuitable for bioassays and biosensors. Later on, several kinds of nanoluminophores with the PRMCECL property in aqueous solutions were synthesized. They included the TiO_2_-TCPP-ABEI (TCPP = 5,10,15,20-tetrakis(4-carboxyphenyl)-porphyrin) nanoluminophore, nGO@TiO_2_ nanoluminophore, and g-C_3_N_4_/ABEI nanoluminophore 189. Furthermore, the applications of these nanoluminophores with the PRMCECL property in label-free ratiometric ECL immunosensors were explored on the basis of the intensity ratio of the potential-resolved ECL signals. However, given that analytes would contribute similar effects to the potential-resolved ECL signals, the use of ratio measurement strategies to obtain improved analytical performance or determine analytes was difficult. Du *et al.* developed a novel ECL nanoluminophore CdSQDs@MOF-5 by encapsulating CdSQDs into MOF-5. Two ECL peaks (ECL-1 and ECL-2) with different colors (with a wavelength of 685 and 475 nm) in the negative potential range (-1.4 and -1.8 V) were generated through the reaction of electroreduced CdSQDs^•-^ and MOF-5^•-^ with SO_4_^•-^. A label-free differential ECL immunosensor was successfully established to detect cTnI with syntropy change in the intensity of ECL-1 and ECL-2, and the synchronized interference that originated from two signals can be eliminated by the subtraction of one ECL signal from the other ECL signal in the potential-resolved ECL emissions 141 (Figure 9C). The third type is ECL-molecule functionalized nanoluminophores. Wang *et al.* synthesized ABEI-functionalized g-C_3_N_4_, which displayed two potential-resolved ECL peaks in the presence of K_2_S_2_O_8_ and H_2_O_2_. On this basis, a metal ion-mediated potential-resolved ratiometric ECL bioassay for the detection of miR-133a was created 190. Similarly, Guo *et al.* successfully synthesized novel ABEI-functionalized GQD ECL nanoluminophores with three completely potential-resolved dual-color ECL emissions and explored their usage in a label-free three potential ratiometric immunosensor for the detection of cTnI with the ECL-1/ECL-3 ratio as the self-correction reference. This work opened a new area in the research on the multicolor ECL of nanoluminophores, which is of great importance in the ECL field from fundamental studies to practical applications 142.

Recently, the interest in QD-based ECL systems has increased because of the large diversity, easy synthesis, extremely efficient and steady signal output, and adjustable photometric properties of QDs 191-193. Li *et al.* 194, for the first time, successfully achieved the multicolor ECL of semiconductor nanocrystals tuned by the size effect by using QDs with a core-shell structure. This approach would provide guidance for the design and preparation of stable and strong multicolor ECL emitters for the simultaneous analysis of multiple components. Li *et al.* 194 systematically studied size-dependent colorful ECL emissions based on core-shell structured CdSe@ZnS QDs in aqueous solutions. The emission color of these QDs was tuned by selecting different sizes of cores. The CdSe@ZnS QDs with different sizes (2.5, 4.0, and 5.9 nm) presented novel colorful ECL emissions at approximately 525, 585, and 625 nm, which were identical to their PL. Similarly, Liu *et al.* 195 generated four sizes of water-dispersible CdS QDs capped with 3-mercaptopropionic acid. Particles emitting green, yellow, pink, and red ECL with positively shifted ECL onset potentials and continuously increasing ECL intensity were created by increasing the particle size of CdS QDs to 1.8, 2.7, 3.2, and 3.7 nm. However, all of the QDs used for ECL production showed significant surface trapping in their emission as evidenced by their shifted and trap-emitting ECL relative to their PL 196-199, resulting in a low PLQY 200-202 and/or multichannel PL decay dynamics 203,204. Comparative studies have shown that ECL is more vulnerable to surface traps on QD surfaces than PL, thus necessitating the creation of a distinct inorganic-organic interface to conceal traps 197,198. Cao *et al.* 205 used the wide bandgap feature of ZnS shells to cover the surface traps on CdSe@CdS QDs, wherein intermediate CdS layers were built to relieve the considerable lattice strain between CdSe and ZnS. Under nonaqueous and aqueous conditions, the effectively synthesized CdSe/CdS/ZnS core/shell/shell QDs showed almost optimal PL characteristics, such as near-unity PLQY (>90%), narrow PL peak, and monoexponential PL decay dynamics. CdSe QDs of various sizes (with average diameters of 5.9 nm (emitting at 549 nm), 6.6 nm (emitting at 592 nm), and 9.0 nm (emitting at 643 nm)) were chosen to obtain spectrally resolved ECL, and their emission wavelengths had to be adjusted to be 50 nm apart from each other. Such an adjustment relies on the regulation of the thickness of the CdS inner shell. The potential-dependent ECL spectra of the green-, yellow-, and red-emitting QDs produced brilliant and steady band-edge ECL that was not only narrow and symmetric but also easily visible and identifiable from one another by the naked eye. Overall, these qualities enabled the presentation of a potentially tunable system for spectrally resolved ECL, demonstrating that the overall ECL would vary its spectrally resolved pattern at various potentials. This approach not only enables detecting several different targets at the same time but also opens up new possibilities for developing multiplex assay.

## Summary and Outlook

The potential-resolved strategy, as a basic methodology for ECL signal output, can guide the design and development of many sensors and has far-reaching academic and practical importance due to its low instrument requirement, short assay time, and good sample throughput. By recounting and discussing its applications in (a) ratiometric ECL sensors, (b) ECL sensors for the simultaneous detection of multiple markers, and (c) multicolor ECL sensors, the design criteria for the particular application mode of the three types of sensors in the potential-resolved strategy were gradually theorized and materialized to facilitate the discovery of new electrochemiluminophores with bespoke properties.

In general, for ratiometric ECL dual-potential biosensors, the most important problem of the traditional ECL-RET and co-reactant competing method is the lack of electrochemiluminescent system pairs for realizing the signal change along with the analyte. Therefore, some improvements in the traditional approach have been achieved, including the development of the dual-role energy transfer intermediate, universal enhancer or quencher, steric hindrance competition strategy, and competitive immunoassay. Studies related to these topics explained the design of sensors and the application of materials on the basis of chemical and electrochemical mechanisms, which are of great importance for guiding the application of theory. In addition, certain novel strategies, such as the enzyme-based ratiometric strategy and the single luminophore ratiometric system, have been found to augment the theoretical basis of the ratiometric ECL method. Furthermore, most previous studies on markers were based on regulating the loaded ECL luminescer, which inevitably increases the complexity of system construction and chemical cross-talk. Therefore, these studies cleverly used the special properties of some co-reactants to control the generation and consumption of ECL co-reactants at different potentials via enzymatic reactions or special chemical reactions. Obtaining resolvable ECL signals within a narrowed potential is the key to increasing the number of the simultaneously detected substances of potentiometric multilabel ECL analysis systems. The main challenges are the limited number of ECL luminophores available and the ineffective control of electrochemical reactions by voltage. Although luminophores with different redox potentials can be easily synthesized, ECL is persistently generated as long as the potential exceeds the corresponding threshold value, which can be compensated for if each luminophore's emission can also be spectrally resolved. In this case, developing a signal probe with a low emission potential and narrow potential is another strategy. As previously stated, the potential-resolved multicolor ECL system in which the color of the combined ECL signal can be adjusted with various voltages can expand the valid potential scale to some extent and also enable the visualization of the detection results. Although these investigations have revealed new possibilities and breakthroughs for ECL analysis, comprehensive research remains to be conducted before the potential-resolved strategy could be extensively applied in the ECL bio-sensing field.

(1) Unique luminophores should be designed to increase data output in a narrow signal window to meet the requirements of clinical high-throughput testing. Several techniques that can provide theoretical possibilities exist: I. The ongoing development of new water-soluble low-potential emitters will improve the utilization of the potential window to achieve the simultaneous detection of biomarkers in the same batch. II. The use of electrochemiluminophores with narrow spectral distributions will enable the resolution of great numbers of emitters over a defined voltage range. III. Given that most ECL luminophores can react with multiple co-reactants to emit at different potentials, a single emitter within a multiple co-reactant system can be constructed for multiplex detection, in which markers can be labeled with multiple co-reactants to solve the problem of luminophore shortage. IV. Moreover, combining potential-resolved approaches with some spatial and/or spectral resolution will enable the performance of a considerably increased number of simultaneous assays within a relatively small detection zone.

(2) A large part of ECL reactions rely on H_2_O_2_ or dissolved oxygen as their co-reactants. However, some unresolved issues limit their clinical applications. These issues include the instability of H_2_O_2_, which makes ensuring that the same concentration of H_2_O_2_ is added in every detection difficult. Moreover, the ultralow catalytic efficiency of dissolved O_2_ for ECL necessitates the development of new methods to increase the amount of active co-reactants on the electrode surface. One strategy is to introduce novel ORR catalysts (such as biomimetic nanozymes and single-atom catalysts) that can generate a large number of ROS *in situ* by catalyzing dissolved oxygen in the solution. These catalysts are often macromolecules with carbon and nitrogen matrix backbones and metal active sites that confer them with the ability for self-enrichment and for binding biological macromolecules as tags through covalent modification, which can lead to the amplification of ECL signals.

(3) With the widespread application of the Internet-of-Things and increasing demand for point-of-care diagnostics, real-time, multiplex, and portable ratiometric ECL biosensors will be further developed and perfected against background of the in-depth exploration of the principle of potential discrimination technology. The early development of potential-resolved multiplex ECL suggests that the greatest benefit from these approaches lies in the development of low-cost portable analytical devices (for example, those based on paper microfluidics, rechargeable batteries, and/or supported by mobile phone technology), in which multiple, simultaneous, but still highly sensitive assays are desired.

Overall, the multisignal output ECL system based on a potential-resolved scheme is a promising and desirable strategy for the creation of diverse ECL sensors. Other preferable ECL materials, the deepened understanding of the design criteria for optimal electrochemical and photophysical properties, and the novel mechanisms of potential resolved ECL methodologies are all still being researched and developed.

## Figures and Tables

**Scheme 1:**
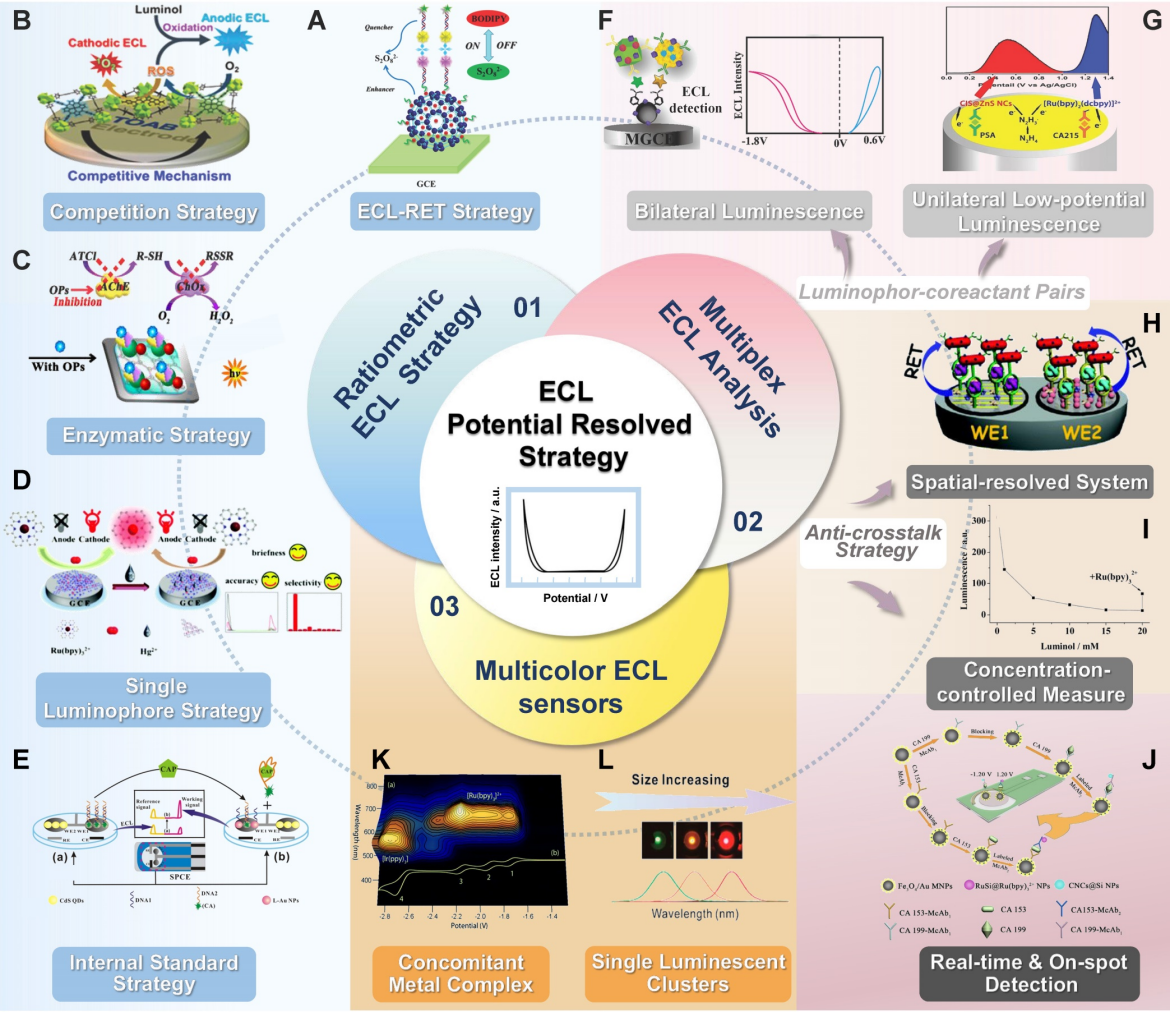
Illustration of the application of potential-resolved strategy in ratiometric ECL sensors based on **(A)** ECL-RET strategy, adapted with permission from 45, copyright 2020 Elsevier B.V., **(B)** competition strategy, adapted with permission from 65, copyright 2020 American Chemical Society, **(C)** enzymatic strategy, adapted with permission from 75, copyright 2017 American Chemical Society, **(D)** single luminophore strategy, adapted with permission from 84, copyright 2020 Royal Chemical Society, **(E)** internal standard strategy, adapted with permission from 89, copyright 2016 Elsevier B.V.; multiplex ECL sensors based on** (F)** bilateral luminescence, adapted with permission from 127, copyright 2020 Elsevier B.V., **(G)** unilateral low-potential luminescence, adapted with permission from 16, copyright 2021 Elsevier B.V., **(H)** spatial-resolved system, adapted with permission from 131, copyright 2019 Royal Chemical Society, **(I)** concentration-controlled measure, adapted with permission from 119, copyright 2014 American Chemical Society, **(J)** real-time & on-spot detection, adapted with permission from 123, copyright 2013 Elsevier B.V.; and multicolor ECL sensors based on **(K)** concomitant metal complex, adapted with permission from 182, copyright 2016 Royal Chemical Society, **(L)** single luminescent clusters, adapted with permission from 205, copyright 2020 American Chemical Society.

**Table 1:** Summary of the partial reports on potential-resolved ratiometric ECL analyses based on the ECL-RET strategy or/and intermediate reagent strategy. Unavailable measurements are represented by "-"

Sensing strategies	Donor	Acceptor	Linear range	LOD	Target	Ref.
RET/intermediate reagent	CdS-CNFs	Luminol-AuNPs	1.0 × 10^-16^-1.0 × 10^-15^ g/mL	3.3 × 10^-14^ g/mL	Carcinoembryonic antigen	57
	CdS NCs	Luminol-AuNPs	-	5.0 × 10^-13^ g/mL	Thrombin	52
	CdS QDs	Au@luminol	1.0 × 10^-5^-1.0 × 10^-2^ M	2.8 × 10^-6^ M	Mg^2+^	30
	g-CdTe QDs	TAEA-Ru	1.0 × 10^-14^-1.0 × 10^-9^ M	2.9 × 10^-12^ M	DA	42
	CdSe/ZnS QDs	Luminol	5.0 × 10^-10^-5.0 × 10^-7^ g/mL	-	Prostate specific antigen	34
	g-C_3_N_4_ NSs	Ag-PAMAM-luminol	2.0 × 10^2^-9.0 × 10^3^ cells/mL	1.5 × 10^2^ cells/mL	HL-60 cancer cells	5
	CdSe/ZnS	Au-Luminol NPs	5.0 × 10^-16^-5.0 × 10^-13^ M	1.2 × 10^-16^ M	Target DNA	46
	CdTe/CdS/ZnS QDs	DNA2/H/Au	5.0 × 10^-12^-1.0 × 10^-8^ M	1.2 × 10^-13^ M	AFB1	39
	GQDs	Luminol-AuNPs	1.0 × 10^-2^-1.0 × 10 U/mL	5.0 × 10^-13^ U/mL	Protein kinase A	24
	CdS NCs	Luminol	5.0 × 10^-16^-1.0 × 10^-14^ M	2.4 × 10^-16^ M	Duplex-specific nuclease	51
RET	O_2_/S_2_O_8_^2-^	PTC-NH_2_	1.0 × 10^-12^- 1.0 × 10^-7^ M	3.5 × 10^-13^ M	Pb^2+^	58
	CdS QDs	Luminol	1.0 × 10^-11^- 2.0 × 10^-6^ M	3.0 × 10^-12^ M	Pb^2+^	59
	MPA-CdS:Eu NCs	Luminol	1.0-3.0 × 10 U/mL	7.0 × 10^-2^ U/mL	Human methyltransferase	60
	CdSe QDs	Luminol	5.0 × 10^-6^-1.0 × 10^-4^ M	5.0 × 10^-6^ M	H_2_O_2_	38
	CdTe@CdS QDs	Luminol	1.0 × 10^-7^-5.0 × 10^-13^ g/mL	4.2 × 10^-15^ g/mL	Thrombin	23
	CdS QDs	Au-Luminol	-	1.3 × 10^-13^ g/mL	Carcinoembryonic antigen	47
	K_2_S_2_O_8_	Fluoroboron dipyrrole	1.0 × 10^-13^-8.5 × 10^-7^g/mL	4.2 × 10^-14^ g/mL	Lactoferrin	45
	HCNS	CPB	1.0 × 10^3^-3.2 × 10^5^ cells/ mL	3.2 × 10^2^ cells/ mL	MCF-7 cells and CD44 receptors	40
	g-C_3_N_4_	PANI/ABEI	1.0 × 10^-13^-4.0 × 10^-11^ g/mL	2.3 × 10^-14^ g/mL	CT	61
	CdS NCs	Luminol	5.0 × 10^-15^-1.0 × 10^-12^M	1.7 × 10^-15^M	mp53 oncogene	36
	Ru-Lu JPs	FAM/Cy5	1.0 × 10^-14^-1.0 × 10^-8^M	8.7 × 10^-15^ /1.2 × 10^-15^ M	miRNA-21/miRNA-155	43
	SnS_2_ QDs@Eu MOFs	L-Au-Pt NRs	1.0 × 10^-11^-1.0 × 10^-7^M	3.2 × 10^-13^ M	KAN	44

**Table 2:** Summary of the partial reports on potential-resolved ratiometric ECL analysis based on the competition strategy. Unavailable measurements are represented by "-"

Competition strategy	Pairs of luminophores	Linear range	LOD	Target	Ref.
H_2_O_2_	LuAuNPs/AuNPs@CNNS	1.0 × 10^2^-1.0 × 10^6^ cells/mL	20 cells/mL	Circulating tumor cells and cell-surface glycans	41
O_2_	Zinc tetrakis [carboxyphenyl]-porphyrin/luminol	2.0 × 10^-4^-10 U /mL	6.5 × 10^-5^ U/mL	PNK	65
	CdTe QDs/ABEI	1.0 × 10^-13^-1.0 × 10^-8^ g/mL	3.0 × 10^-14^ g/mL	Concanavalin A	2
	DBAE/lucigenin	1.0 × 10^-14^-1.0 × 10^-8^ g/ml	3.3 × 10^-15^ g/mL	Human epididymis protein 4	67
H_2_O_2_	CdS QDs/luminol	1.0 × 10^-12^-1.0 × 10^-10^ g/ mL	6.2 × 10^-13^ g/mL	Carcinoembryonic antigen A	33
	ABEI/g-C_3_N_4_	1.0 × 10^-13^-1.0 × 10^-8^ g/mL	3.3 × 10^-14^ g/mL	Zearalenone	63
Steric hindrance	TiO_2_-Ru(bpy)32+ NPs/PEI-CdS QDs	1.0 × 10^-9^-1.0 × 10^-4^ M	1.7 × 10^-11^ M	SA	68
	nGO@TiO_2_NLPs/K_2_S_2_O_8_	1.0 × 10^-13^- 1.0 × 10^-10^ M	4.0 × 10^-14^ M	cTnI	69
	Au@CDs NFs/Au@luminol NPs	1.0 × 10^-15^-1.0 × 10^-11^ M	3.4 × 10^-16^ M	p53 DNA	70
Competitive immunoreaction	Luminol/CS-AgI	1.0 × 10^-15^-2.0 × 10^-8^ g/mL	1.0 × 10^-15^ g/mL	AFP	72
H_2_O_2_	Ir NRs/CdS QDs	5.0 × 10^-12^-5.0 × 10^-8^ M	1.67 × 10^-12^ M	EP	73
O_2_	Luminol/Cu-TCPP [Zn]	5.0 × 10^-3^-5.0 U/mL	3.7 × 10^-3^ U/mL	Protein kinase A	49

**Table 3:** Summary of the partial reports on potential-resolved ratiometric ECL analysis based on enzymes. Unavailable measurements are represented by "-"

Enzyme	Pairs of luminophores	Linear range	LOD	Target	Ref.
AChE	rGO-CdTe QDs/PFO dots	5.0 × 10^-13^-1.0 × 10^-8^ M	1.25 × 10^-13^ M	OPs	75
	c-PFBT NPs/L-CdS QDs	5.0 × 10^-13^-5.0 × 10^-7^ M.	1.25 × 10^-13^ M	EP	81
XOD	rGO-CdTe QDs]/luminol	2.0 × 10^-11^ -2.0 × 10^-3^ M	7.0 × 10^-12^ M	Hx	63
HRP	ABEI/GSH	1.0 × 10^-13^ -1.0 × 10^-12^ g/mL	3.3 × 10^-14^ g/mL	ZEN	71
CS-AgI	Luminol/K_2_S_2_O_8_/CS-AgI	1.0 × 10^-15^-2.0 × 10^-8^ g/mL	1.0 × 10^-15^ g/mL	AFP	82
GOx	PFO NPs	5.0 × 10^-17^-1.0 × 10^-10^ M	1.7 × 10^-17^ M	miRNA-155	80

**Table 4:** Summary of the partial reports on potential-resolved ratiometric ECL analysis based on single luminophores. Unavailable measurements are represented by "-"

Luminophor	Co-reactant	Linear range	LOD	Target	Ref.
Ru(bpy)32+	Au-g-C_3_N_4_ NSs	5.0 × 10^-10^-5.0 × 10^-7^ M	2.0 × 10^-10^ M	Hg^2+^	84
NGQDs	O^2·-^/HO^2-^	1.0 × 10^-3^-7.0 × 10^-2^ M	2.0 × 10^-4^ M	Co^2+^	85
g-C_3_N_4_	K_2_S_2_O_8_/TEA	3.0 × 10^-16^-1.0 × 10^-11^ g/mL	1.0 × 10^-16^ g/mL	AFP	53
Ru(bpy)32+	C-dots	1.0 × 10^-9^-1.0 × 10^-4^ M	4.7 × 10^-10^ M	TC	87

**Table 5:** Summary of the partial reports on potential-resolved ratiometric ECL analysis based on the internal standard strategy. Unavailable measurements are represented by "-"

Luminophor	Tag	Linear range	LOD	Target	Ref.
**Internal standard strategy based on a single electrode**					
L-AuNPs/CdS NWs	cTnI-rGO-AuNPs-CAT	5.0 × 10^-13^-1.0 × 10^-7^ g/mL	1.0 × 10^-13^ g/mL	cTnI	89
L-AuNPs/CdS QDs	CA	1.0 × 10^-10^-1.2 × 10^-7^M	3.0 × 10^-11^ M	CAP	90
**Internal standard strategy on double disk electrode**					
Substrate/internal reference	Label emitter	Linear range	LOD	Target	Ref.
Luminol	CdTe QDS	1.0 × 10^2^-6.5 × 10^3^ cell/mL	80 cell/mL	MCF-7 cells	93
Au@Luminol	CIZS/ZnS QDs	1.0 × 10^-14^-1.0 × 10^-9^ M	1.82 × 10^-15^ M	Thrombin	94
g-C_3_N_4_	Pdots	1.0 × 10^-14^-1.0 × 10^-8^ g/mL	3.3 × 10^-15^ g/mL	Human epididymis protein 4	32

**Table 6:** Summary of partial reports on potential-resolved multiplex analysis by ECL technology. Unavailable measurements are represented by "-"

	ECL probe & onset potential	Target	LOD	Linear range	Ref.
**Bilateral anodic-and-cathodic-potential luminescence**					
1	CdTe@CdS QDs; -1.12 V	AFP	1.0 × 10^-16^ g/mL	2.5 × 10^-16^-2.0 × 10^-11^ g/mL	121
	Luminol; +0.6 V	CEA	1.0 × 10^-16^ g/mL	2.5 × 10^-16^-2.0 × 10^-11^ g/mL	
2	CdS QDs; -1.15 V	MG	3.0 × 10^-11^ M	1.0 × 10^-10^ -1.0 × 10^-7^ M	117
	L-AuNPs; +0.6 V	CAP	7.0 × 10^-11^ M	2.0 × 10^-10^ -1.5 × 10^-7^ M	
3	Ru-NH_2_; 1.25 V	CA125	4.0 × 10^-4^ U/mL	0.001-100 U/mL	124
	AuNPs/g-C_3_N_4_; -1.3 V	SCCA	3.3 × 10^-13^ g/mL	1.0 × 10^-12^-1.0 × 10^-7^ g/mL	
4	AuNPs/luminol; +0.6 V	CEA	-	3.3 × 10^-9^-1.6 × 10^-8^ g	125
	Ru(bpy)32+; -1.0V	AFP	-	2.0 × 10^-10^ -1.1 × 10^-9^ g	
5	Luminol; 0.6 V	Acetamiprid	1.5 × 10^-14^ M	1.0 × 10^-10^ -1.0 × 10^-13^ M	101
	g-C_3_N_4_; -1.5 V	Malathion	1.8 × 10^-14^ M	1.0 × 10^-10^ -1.0 × 10^-13^ M	
6	DMSA-CdTe QDs; -0.89 V	AFP	1.0 × 10^-12^ g/mL	1.0 × 10^-12^-2.0 × 10^-8^ g/mL	115
	TiO_2_-GSH-CdTe QDs; -1.25 V	AFP-L3	3.2 × 10^-12^ g/mL	3.2 × 10^-12^-3.2 × 10^-8^ g/mL	
7	g-C_3_N_4_@AuNPs; -1.4 V	miRNA-141	3.0 × 10^-16^ M	1.0 × 10^-15^ -1.0 × 10^-11^ M	126
	Ru-MOF;+1.5 V	miRNA-21	3.0 × 10^-16^ M	1.0 × 10^-15^-1.0 × 10^-11^ M	
8	AuPNs/PDI; -0.6 V	CEA	7.3 × 10^-14^ g/mL	1.0 × 10^-13^g/mL-1.0 × 10^-9^ g/mL	114
	AuPNs/luminol; +0.6 V	AFP	5.6 × 10^-14^ g/mL	1.0 × 10^-13^g/mL-1.0 × 10^-9^ g/mL	
9	Luminol; +0.6 V	IFN-γ	1.6 × 10^-12^ g/mL	1.6 × 10^-12^-2.0 × 10^-10^ g/mL	116
	Carbon QDs; -1.8 V	TNF-α	1.6 × 10^-12^ g/mL	1.6 × 10^-12^-2.0 × 10^-10^ g/mL	
	CdS QDs; -1.2 V	IL-2	1.6 × 10^-12^ g/mL	1.6 × 10^-12^-2.0 × 10^-10^ g/mL	
10	Au @ BSA MSs-luminol; 0.32 V	2,6-Sialylated glycans	3.3 × 10^-15^ g/mL	1.0 × 10^-14^-1.0 × 10^-2^ g/mL	127
	TZZ; -1.8 V	2,3-Sialylated glycans	2.1 × 10^-15^ g/mL	1.0 × 10^-14^-1.0 × 10^-2^ g/mL	
11	Ru(bpy)32+; +1.2 V	AFP	2.0 × 10^-11^ g/mL	-	128
	Carbon nanodots; -1.2 V	CA153	5.0 × 10^-3^ U/mL	-	
		CA199	6.0 × 10^-3^ U/mL	-	
		CEA	4.0 × 10^-12^ g/mL	-	
12	Ru [phen]_3_^2+^; +1.2 V	MCF-7 cancer cells	15	1.0 × 10^2^-1.0 × 10^6^ cells/mL	129
	Concanavalin A-conjugated AuNP-modified graphite-C_3_N_4_; -1.6 V	N-glycan expression	-	-	
**Unilateral low-potential luminescence**					
	ECL probe and onset potential	Target	LOD	Linear range	Ref.
13	[dfppy)_2_Ir(dcbpy]PF_6_; +1.4 V	MMP-2	5.0 × 10^-9^ g/mL	1.0 × 10^-8^-3.0 × 10^-7^ g/mL	7
	[Ru [bpy]_2_ [mcbpy-O-Su-ester] [PF6]_2_; +0.9 V	MMP-7	1.0 × 10^-11^ g/mL	5.0 × 10^-11^-1.0 × 10^-9^ g/mL	
14	CIS@ZnS NCs; 0.10 V	PSA	-	-	130
	[Ru [bpy]_2_ [dcbpy]]^2+^; 1.06 V	CA125	-	-	
**Other miscellaneous potential-resolved luminescence**					
	ECL probe and onset potential	Target	LOD	Linear range	Ref.
15	CdS nanowires; -	Myo	2.0 × 10^-13^ g/mL	5.0 × 10^-13^-5.0 × 10^-7^ g/mL	131
	RuSi@ Ru(bpy)32+ NPs; -	cTnI	5.0 × 10^-13^ g/mL	1.0 × 10^-12^ -1.0 × 10^-7^ g/mL	
16	Luminol-AuNPs; -	Adenosine	2.2 × 10^-12^ M	5.0 × 10^-12^-5.0 × 10^-9^ M	132
	ABEI-AuNPs: -	Thrombin	1.2 × 10^-14^ M	5.0 × 10^-14^ -5.0 × 10^-10^ M	
17	Au@luminol;	MCF-7 cells	20	1.0 × 10^2^ -1.0 × 10^6^ cells/mL	133
	CdS QDs;	Mannose	-	1.0 × 10^-13^-1.0 × 10^-12^ M	
		EGFR	-	1.0 × 10^-10^-1.0 × 10^-9^ g/mL	

## Abbreviations

ECL: electrochemiluminesence
MIA: multiplex immunoassay
ECL-RET: electrochemiluminescence resonance energy transfer
Ru(bpy)32+: Tris(2,2'-bipyridine)Ru(II)
HRP: Horseradish peroxidase
PAMAM: Poly(amidoamine)
CdS QDs: CdS quantum dots
Cy5: cyanine dye
PL: photoluminescence
CPB: CsPbBr_3_ nanocrystals
Rh6G: rhodamine 6G
NPs: nanoparticles
NFs: nanoflowers
Cu_2_O: cuprous oxide
FCA: ferrocenecarboxylic acid
HCNS: hollow graphitic carbon nitride nanospheres
ORR: oxygen reduction reaction
PNK: polynucleotide kinase
ABEI: *N*-(aminobutyl)-*N*-(ethylisoluminol)
DBAE: 2-(dibutylamino)ethanol
AuNPs: gold nanoparticles
MOF: metal-organic framework
Cu-TCPP(Zn): copper-based zinc porphyrinic
AFP: alpha fetoprotein
PFO: poly(9,9-dioctylfluorene)
Hx: hypoxanthine
AChE: acetylcholinesterase
GSH: glutathione
ZEN: Zearalenone
NGQD: nitrogen-doped graphene quantum dots
TEA: triethanolamine
CNTs: carbon nanotubes
cTnI: cardiac troponin I
SPCE: screen-printed carbon electrode
CAP: chloramphenicol
CdS NWs: CdS nanowires
L-AuNPs: luminol-gold nanoparticles
DDCE: dual-disk glassy carbon electrode
PSA: prostate specific antigen
CA: chlorogenic acid
g-C_3_N_4_: carbon nitride
Pdots: polymer dots
L012: 8-amino-5-chloro-7-phenylpyrido(3,4-d)pyridazine-1,4(2H,3H)-dione
Con A: concanavalin A
PDI: *N*,*N*-bis-(3-dimethylaminopropyl)-3,4,9,10-per tetracarboxylic diimide
DMSA-CdTe: 2,3-dimercapto-succinic acid-stabilized CdTe
ITO: indium tin oxide
LTBI: latent tuberculosis infections
MG: malachite green
μ-PADs: microfluidic paper-based analytical devices
FCA: ferrocenecarboxylic acid
MIP: molecularly imprinted platform
nGO@TiO_2_NLPs: nanoluminophore nanographene oxide wrapping titanium dioxide
PAMAM-CIZS/ZnS QDs: PAMAM-CuInZnS/ZnS quantum dots
NSs: nanosheets
CO: 7-(diethylamino) coumarin-3-carboxylic acid
NA: 3-(4-amino-1,8-naph-thalimid) propanoic acid
OLEDs: organic light-emitting diodes
SECLDs: solid-state electrochemiluminescence devices
CS-AgI: chitosan-functionalized silver iodide
JPs: Janus particles
DA: Dopamine
AFB1: Aflatoxin B1
CT: calcitonin
CS-AgI: chitosan functionalized silver iodide
PNK: polynucleotide kinase
Con A: concanavalin A
HE4: human epididymis protein 4
SA: Sialic acid
cTnI: cardiac troponin I
EP: ethyl paraoxon
PKA: protein kinase A
c-PFBT NPs: carboxyl-functionalized poly((9,9-dioctylfluorenyl-2,7-diyl)-co-(1,4-benzo-thiada-zole)) nanoparticles
L-CdS QDs: L-cysteine capped CdS quantum dots
OPs: organophosphorus pesticides
Hx: hypoxanthine
CA: chlorogenic acid
TB: thrombin
